# Engineering *Rhodosporidium toruloides* for limonene production

**DOI:** 10.1186/s13068-021-02094-7

**Published:** 2021-12-22

**Authors:** Sasa Liu, Mengyao Zhang, Yuyao Ren, Guojie Jin, Yongsheng Tao, Liting Lyu, Zongbao K. Zhao, Xiaobing Yang

**Affiliations:** 1 College of Enology, Northwest A&F University, Yangling, Shaanxi 712100 People’s Republic of China; 2 Laboratory of Biotechnology, Dalian Institute of Chemical Physics, Chinese Academy of Sciences, 457 Zhongshan Road, Dalian, 116023 People’s Republic of China

**Keywords:** Monoterpene, Limonene, Microbial production, Oleaginous yeast, Metabolic engineering

## Abstract

**Background:**

Limonene is a widely used monoterpene in the production of food, pharmaceuticals, biofuels, etc. The objective of this work was to engineer *Rhodosporidium toruloides* as a cell factory for the production of limonene.

**Results:**

By overexpressing the limonene synthase (LS), neryl pyrophosphate synthase (NPPS)/geranyl pyrophosphate synthase and the native hydroxy-methyl-glutaryl-CoA reductase (HMGR), we established a baseline for limonene production based on the mevalonate route in *Rhodosporidium toruloides*. To further enhance the limonene titer, the acetoacetyl-CoA thiolase/HMGR (EfMvaE) and mevalonate synthase (EfMvaS) from *Enterococcus faecalis*, the mevalonate kinase from *Methanosarcina mazei* (MmMK) and the chimeric enzyme NPPS-LS were introduced in the carotenogenesis-deficient strain. The resulting strains produced a maximum limonene titer of 393.5 mg/L.

**Conclusion:**

In this study, we successfully engineered the carotenogenesis yeast *R. toruloides* to produce limonene. This is the first report on engineering *R. toruloides* toward limonene production based on NPP and the fusion protein SltNPPS-CltLS. The results demonstrated that *R. toruloides* is viable for limonene production, which would provide insights into microbial production of valuable monoterpenes.

**Supplementary Information:**

The online version contains supplementary material available at 10.1186/s13068-021-02094-7.

## Background

Limonene is a widely used natural monoterpene in food, beverage, cosmetics, biomaterials, pharmaceuticals, and advanced biofuels [1–3]. It stands also as a versatile platform chemical that can be decorated to form various value-added fine chemicals, including linalool, carveol, menthol, limonene-1,2-epoxide, α-terpineol, perillyl alcohol, perillic acid and limonene-1,2-diol, by various natural or engineered microorganisms, and chemical processes [4, 5]. Owing to its widespread applications, the market size for limonene is expected to reach 1.9 billion US dollars (9–10$ per kilogram) [6, 7]. However, current industrial production of limonene is unsustainable in that the plant-based extraction suffers from limited feedstock variability while the chemical synthesis is unfavored due to its high energy input, environmental concerns, and toxic impurities [8].

Limonene produced through microbial technology can be regarded as a natural alternative to that extracted from plants. With the development of metabolic engineering and synthetic biology, microbial biosynthesis offers a feasible option to produce monoterpenes [3]. To date, microbes including *E. coli* [3, 9], *S. cerevisiae* [10–13], *Y. lipolytica* [1, 8] and cyanobacteria [5, 14] have been engineered to produce limonene by recruiting their 2-methyl-d-erythritol-4-phosphate (MEP) pathway and/or the mevalonate (MVA) pathway with geranyl diphosphate (GPP) and/or neryl diphosphate as the direct precursor (Table 1). However, the highest titer was only 3.6 g/L obtained with engineered *E. coli*, which was far from industrial production [7, 9, 15].

**Table 1:** Limonene titers in different hosts

Host	Limonene synthase	Stereo isomer	Pathway	Direct precursor of LS	Carbon source	Cultivation mode	Titer (mg/L)	Productivity (mg/L/h)^a^	Yield (mg/g)	Reference
*Escherichia coli* BL21 (DE3)	*Mentha spicata*	S	MEP&MVA	GPP	Glycerol	Bioreactor Fed-batch	3630	151	< 10^b^	Rolf et al. [9]
*Saccharomyces cerevisiae* EGY48	*Citrus limon*	R	MVA	GPP	Glucose	Bioreactor Fed batch	2580	3.36^b^	NA	Dusséaux et al. [11]
*Yarrowia lipolytica* Po1f	*Agastache rugosa*	R	MVA	NPP	Glycerol &Citrate	Bioreactor; Fed-batch	165.3	1.15^a^	41.3^b^	Cheng et al. [8]
*Synechococcus elongatus* UTEX 2973 (mutated)	*Mentha spicata*	S	MEP	GPP	CO2;	250 μmol photons/m^2^/s	16.4	0.34	NA	Lin et al. [14]
*Rhodosporidium toruloides* IFO0880	*Abies grandis*	S	MVA	GPP	Glucose	Shake flask batch	Trace	NA	NA	Zhuang et al. [20]
*Rhodosporidium toruloides* np11	*Citrus limon*	R	MVA	NPP	Glucose	5-mL test-tube; batch	393.5	0.66	19.675	This work

*NA* not available

^a^Calculated by the data presented in the references

^b^Calculated by the data estimated from the figure or graph

*Rhodosporidium toruloides* represents an emerging robust producer for lipids and a native host for carotenoids, which is capable of consuming a wide range of carbon sources while tolerating the toxic byproducts in lignocellulose hydrolysates [16]. The carotenogenic *R. toruloides* has been engineered to produce various terpenoids such as *β*-carotene, ent-kaurene, bisabolene and amorphadiene via recruiting its native MVA pathway [17–20]. In a recent attempt, *R. toruloides* was rewired for producing monoterpenes; unfortunately, only trace amount of limonene was produced [20], which might be attributed to the common challenges including insufficient supply of precursor, lack of efficient enzyme, tight intracellular self-regulation, and the cytotoxicity of limonene [21].

The sufficient supply of GPP or NPP is most essential for efficient microbial synthesis of limonene. However, like most microbes, *R. toruloides* does not have a specific GPP synthase (GPPS), and instead employs the farnesyl diphosphate synthase (FPPS), which produces mainly farnesyl diphosphate and tiny amount of GPP, for supporting cell growth [22]. There are two strategies for boosting monoterpene direct precursor pool: (1) employing an efficient GPPS or engineering the FPPS to generate more GPP; (2) constructing an orthogonal pathway based on nerol diphosphate (NPP) [21]. The GPP-based limonene biosynthesis usually encounters endogenous competition and native regulation in the host while the NPP-dependent route is theoretically independent of the natural metabolic machinery and thus may have potential for high-level production [21].

**Fig. 1:**
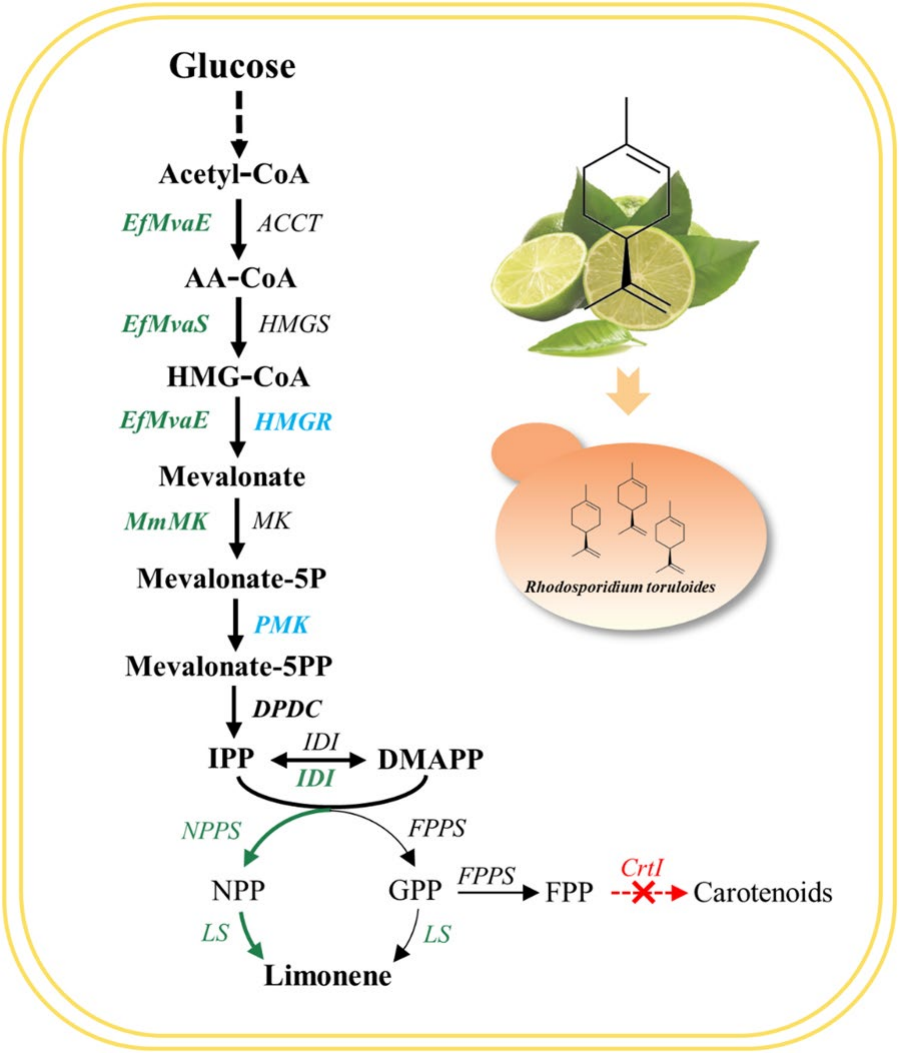
Biosynthetic pathways for bioproduction of limonene within *R*. *toruloides*

Therefore, we established an orthogonal R-limonene biosynthesis platform in *R. toruloides* on NPP-based route (Fig. 1)*.* To boost the limonene production, we employed the native 3-hydroxy-3-methy-glutaryl CoA reductase (HMGR), introduced the efficient upper MVA module from *Enterococcus faecalis*, screened LS from eight sources, and employed the *Solanum lycopersicum* originated neryl diphosphate synthase 1 (SltNPPS1). We further improved limonene titer by implanting the limonene-generating module into a carotenogenesis-deficient *R. toruloides* strain, and reduced the metabolic flux leakage by using the chimeric SltNPPS1-CltLS1. The final strains with an optimal pathway were fermented in a biphasic system with dodecane as the overlay, and received a maximum R-limonene titer of 383.5 mg/L. Our results demonstrated that *R. toruloides* is a potential host for economic limonene production, which would also provide insights into microbial production of other valuable monoterpenes.

## Results and discussion

### Construction of limonene biosynthesis baseline with *R. toruloides*

**Fig. 2:**
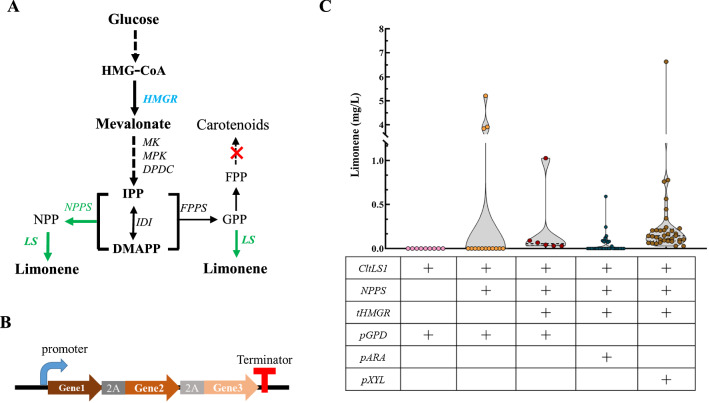
Construction of baseline for limonene production with *R. toruloides* np11

Currently, owing to the lack of genetic tools, it remains challenging to rewire *R. toruloides* when multiple genes are required to be targeted. 2A peptide-mediated multiple protein co-production enables up to four genes overexpression as a single operon, driven under only one promoter [23]. Importantly, the use of 2A peptide sequences is free from imbalanced protein expression and the separation of genes located between 2A peptide sequences is nearly 100%. Their small size (18–22 amino acids) and divergent amino sequences can minimize the chance for homologous recombination [23]. Since it involves multiple enzymes for constructing an efficient limonene biosynthetic pathway based on NPP (Fig. 2A; Additional file 1: Fig S1), we employed the 2A peptide-mediated multiple proteins production, which has been demonstrated successful in building a carotenoids biosynthesis route (Fig. 2B) [24].

GPP and NPP are the direct precursors for monoterpenes, which are biosynthesized by condensing isopentenyl diphosphate (IPP) and dimethylallyl diphosphate (DMAPP) [21]. Sufficient accumulation of GPP or NPP is essential for the overproduction of downstream products. However, as for other yeast, *R. toruloides* does not have an efficient GPPS or NPPS to support monoterpene biosynthesis [25]. Instead, GPP is generated at very low level under the catalysis of the native FPPS to maintain cell growth. Thus, no limonene was detected when only the *CltLS1* (AAM53944.1) was overexpressed under the control of the strong constitutive glyceraldehyde-3-phosphate dehydrogenase promoter (pGPD) (Fig. 2A), even the CltLS1 was previously documented more effective than the one from *M. spicata* [26]. Our result was constant with the previous study that only trace amount of limonene was produced by simply introducing a LS [20]. In addition to the inadequate pool, using GPP for limonene biosynthesis would encounter the competing pathways within *R. toruloides* since it involved in many downstream chemicals biosynthesis, and is rigorously regulated by the cellular metabolic systems [27]. Thus, we shift to constructing NPP-based orthogonal limonene biosynthetic pass.

NPP was reported more efficient toward supporting limonene biosynthesis in *Y. lipolytica*, *S. cerevisiae* and *E. coli* [3, 10, 28, 29]. We thus introduced *S. lycopersicum* NPP synthase 1 (SltNPPS1) (truncated at the position of S4) to provide NPP as the precursor for CltLS1 (AAM53944.1) toward limonene production. However, only 3 of the 30 selected transformants harboring CltLS1and SltNPPS1 produced limonene (Fig. 2C), which might be attributed to the insufficient pool of mevalonate, IPP and DAMPP in *R. toruloides.* Besides, the random integration sites of target genes on the chromosome influenced the expression efficiency that affected few producers [30].

Mevalonate is the most critical intermediate metabolite in the MVA pathway used for the biosynthesis isoprenoids [31]. The HMGR is the key knot controlling enzyme in the MVA pathway where overexpression of the key genes in MVA pathway is widely adopted in boosting precursor supply. We thus co-expressed the native tHMGR with CltLS and SltNPPS (NLH module) promoted by pGPD, and the obtained strains produced limonene reached up to 1.03 mg/L in 50 mL test tubes after 120 h cultivation in YPD medium (Fig. 2C). The average titers were around 0.18 mg/L while the middle values were about 0.04 mg/L (Fig. 2). The ATMT mediated transformation usually results in the random integration of the heterologous genes into the *R. toruloides* genome, and usually with just one copy [30], which may be unfavorable for overproducing target products. We thus put the limonene-producing module NLH under the xylose reductase promoter (pXYL) and l-arabitol 4-dehydrogenase promoter (pARA) with the aim of elevating the limonene titer (Fig. 2B) [32]. Then we cultivated the obtained strains with glucose, xylose and arabinose as the carbon source (Additional file 2: Table S1). However, most of the obtained limonene titers were comparable to those obtained with the strains with pGPD as the promoter (Additional file 2: Table S1) while the transformants under the control of previously reported strong pXYL affected a maximum titer of 6.64 mg/L and an average of 0.40 mg/L. The results here indicated that the limonene production is influenced by the efficiencies of LS catalysis, precursor supply and promoter driving.

Interestingly, in our study, although most of the reconstructed strains produced limonene as the main product, some generated more relevant monoterpenes than limonene. In particular, some engineered strains produced up to 6 monoterpenoids in addition to limonene (Additional file 1: Fig S2). The variance of product profile might be attributed to the substrate conformation that *M. spicata* LS produced more byproducts when using NPP as the precursor [33].

### Production of limonene with carotenogenesis-deficient *R. toruloides*

**Fig. 3:**
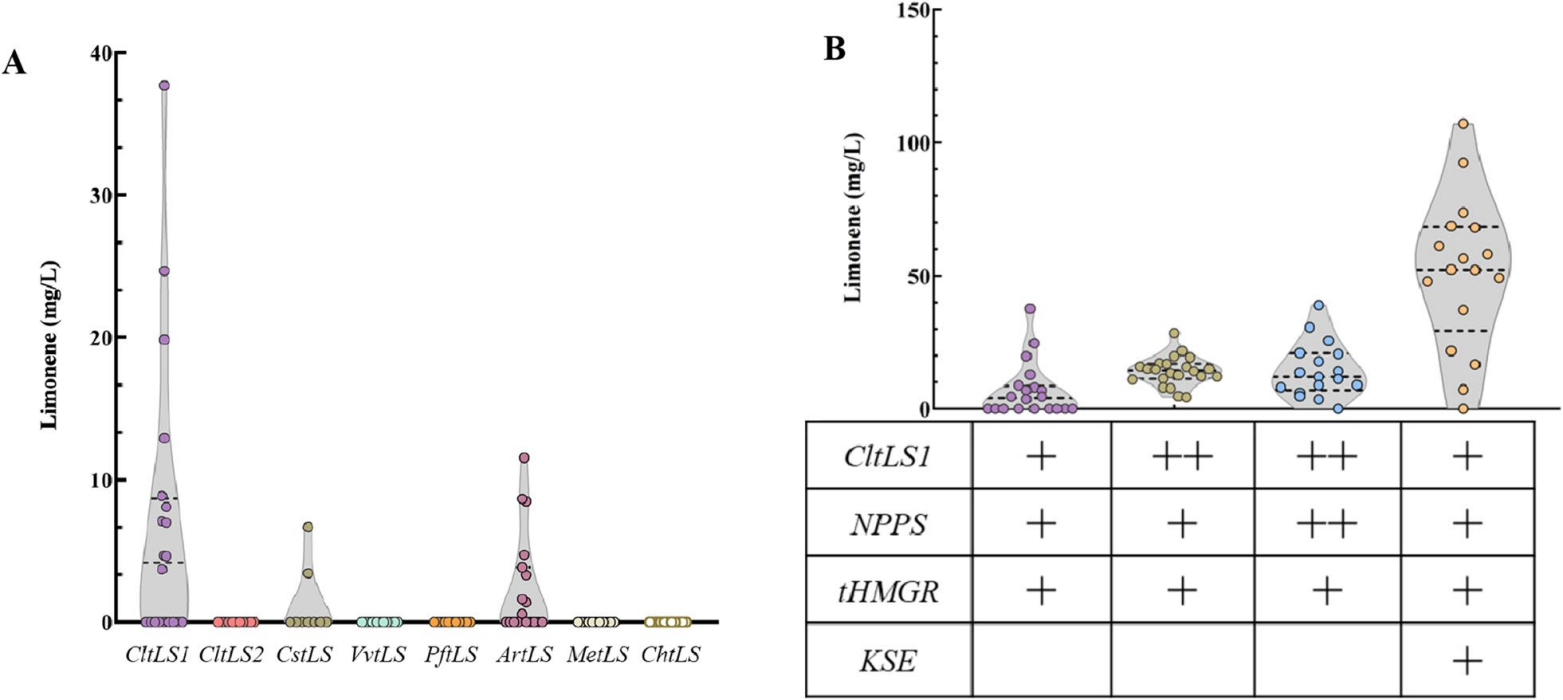
Limonene production with carotenogenesis deficient *R. toruloides*

Blocking the competitive pathways is often deployed to boost the bioproduction of target chemicals. As a native carotenoids producer, *R. toruloides* was reported to produce more than 2 mg carotenoids per g dry cell weight [24]. To further boost the titers, we thus transplanted our pXYL-driven limonene-producing module NLH into a carotenogenesis-deficient strain [34], where the maximum R-limonene titer was 37.71 mg/L (Lim 16-6), and the average 6.69 mg/L in 50-mL test tubes, representing 6-, and 44.6-fold increase compared to those in wild type *R. toruloides* NP11 (Fig. 3C; Additional file 1: Fig S3). These results demonstrated that the native carotenogenesis path presents a tough competition against limonene overproduction in *R. toruloides*.

Although the limonene titers have been significantly improved via employing the *Δ*Crt host, there are still two possible limiting factors. One is inefficiency of the LS, the other is the limited metabolites flux of MVA module. Therefore, LSs from *Agastache rugosa* (*ArtLS*), *Chamaecyparis obtusa* (*ChtLS*), *Citrus limon* (*CltLS*1and *CltLS*2), *Citrus sinensis* (*CstLS*), *Mentha piperita* (*MptLS*), *Perilla frutescens* (*PftLS*), and *Vitis vinifera* (*VvtLS*) were overexpressed in the carotenogenesis invalid strain to investigate their efficiency toward limonene production (Fig. 3; Additional file 1: Fig S4). Notably, all the N-terminal transit domains were removed before they hamper the correct folding and maturation of LS in *R. toruloides* (Additional file 1: Fig S2B) [6, 35, 36]. However, only the reconstructed strains carrying the LSs from *A. rugose* and *C. sinensis* produced limonene, where the highest titers were 11.6 mg/L and 6.72 mg/L, respectively (Fig. 3C). The results herein demonstrated that the CltLS1 was the most efficient enzyme in terms of limonene biosynthesis based on NPP in *R. toruloides,* and further improvement was conducted using the CltLS1. We then introduced an additional copy of CltLS or CltLS1&SltNPPS1 to the strain Limi16. The resulting averaged titers were improved from 6.69 mg/L to 14.22 mg/L and 15.45 mg/L, indicating that the catalysis efficiency of LS is critical for the limonene overproduction (Fig. 3C). However, no significant improvement was observed in terms of the highest limonene titers with the obtained strains, which might be attributed to the insufficient precursor pools for mevalonate, IPP and DMAPP.

*Enterococcus faecalis*-originated acetyl-CoA acetyltransferase/HMG-CoA reductase (EfMvaE) and HMG-CoA synthase (EfMvaS) were reported more efficient than other sourced enzymes for biosynthesis of mevalonate while *Methanosarcina mazei*-derived mevalonate kinase (MmMK) was reported free from DMAPP and GPP feedback inhibition and 5 times more efficient than that from *S. cerevisiae* [37, 38]. Additionally, EfMvaE, EfMvaS and MmMK (the KSE module referred in this study) have been documented for sufficiently facilitating limonene production in *E. coli* [3]. Therefore, to further increase the biosynthesis of mevalonate from acetyl-CoA, we overexpressed the KSE module in *R. toruloides*. As shown in Fig. 3C, the resulted strains carrying the spare MVA module significantly increased the limonene titers, of which the highest was 107.23 mg/L (Lim16-8, Additional file 2: Table S1) and the averaged was 54.43 mg/L, representing 2.8- and 12-times improvement over the group without the KSE module. Hence, enhancing the upstream in the MVA route while blocking the carotenogenesis contributed to the improved limonene titers. These results might provide reference for revealing the major limiting steps toward limonene production.

### Improving limonene production via recruiting fusion proteins

**Fig. 4:**
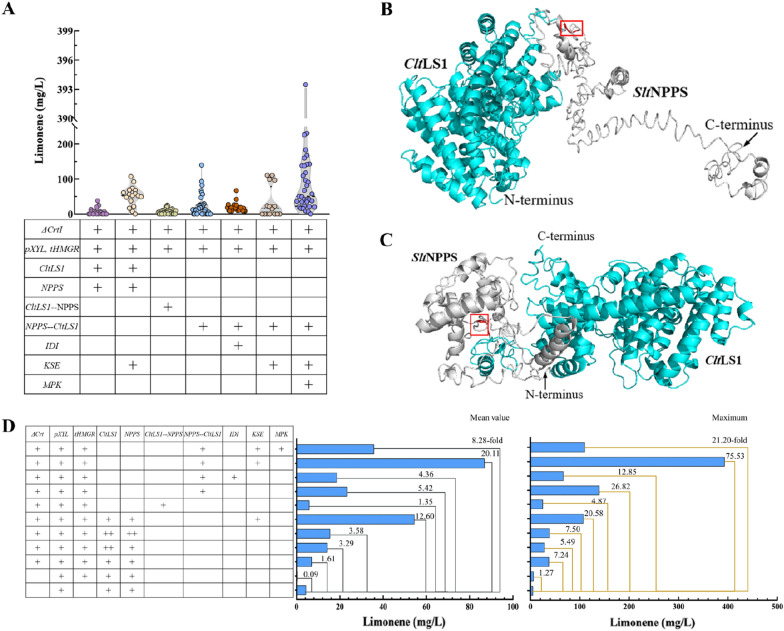
The influence of gene fusion and MVA pathway manipulation on limonene production

The metabolic flux leakage in the biosynthesis pathway may impede the efficiency of limonene production. Protein fusion is a widely applied strategy for enzyme modification, especially the proteins catalyzing sequential reactions, since it facilitates substrate channeling, reduces intermediates loss and improves enzyme activity and stability. The protein fusion strategy has been successfully utilized in boosting the production of terpenoids, such as the sesquiterpene farnesene and the diterpene miltiradiene [39]. Flexible linkers like GGGS are often employed when fusing proteins to relieve folding interference between proteins and enable the manipulated proteins to maintain their native activity. With the hypothesis that similar molecular reactions may also exist between NPPS and LS, we fused the protein CltLS1 and the SltNPPS in different orders by using the “GGGS” linker (Fig. 4) [40] and introduced the chimeric proteins into the ΔCrt *R. toruloides* strain. The transformants expressing the fused protein CltLS1-SltNPPS1 produced a maximum of 25.36 mg/L (strain Lim 34) of limonene, almost even amount as the one carrying separate enzymes of CltLS1 and SltNPPS1, while the strains carrying the SltNPPS1-CltLS1 protein affected a maximum of 139.74 mg/L (strain Lim11-26), representing a 5.5-fold increase compared to strain Lim 34 (Additional file 2: Table S1). The results indicated that the SltNPPS1-CltLS1 fusion outperforms the CltLS1-SltNPPS1 as well as separate expression of CltLS1 and SltNPPS1 in considering the limonene production.

All the fusion proteins constructed with SltNPPS and CltLS were initially expected to present a superior kinetic property in delivering NPP toward CltLS by avoiding free diffusion of the intermediate into the cytoplasm. In addition, higher substrate channeling efficiency was also anticipated to reduce the NPP flux leakage into potentially competing metabolic pathways. However, the SltNPPS1-CltLS1 fusion was found significantly more effective in limonene production than that of CltLS1-SltNPPS1 (Fig. 4). To explore the reason for the differences, we conducted 3D models of the proteins using the web-based tool [41]. The schematic diagram indicated that the distance between the two catalysis domains of SltNPPS and CltLS in the SltNPPS1-CltLS1 fusion version is shorter than that of CltLS1-SltNPPS1. In addition, the modeling results showed that the CltLS1-SltNPPS1 (especially the SltNPPS1 domain) has a loosely coupled architecture while the SltNPPS1-CltLS1 displays a more compact one. Since the mature NPPS works as a dimer, its regular arrangement in the chimeric SltNPPS1-CltLS1 may result in an increase the biosynthesis efficiency and local concentration of NPP that available for limonene synthesis. This might be the reason why the SltNPPS1-CltLS1 fusion carrying strains showed higher limonene titers. To further enhance the limonene titers, we overexpressed the KSE module in the SltNPPS1-CLtLS1 carrying strain Lim 11, the resulting transformants yielded a maximum limonene titer of 393 mg/L (Fig. 4D), which represented 20- and 75-fold increase in averaged and maximum titers, respectively. We further introduced IDI from *Phaffia rhodozyma*, and the native MPK, by fusing them to the C-terminal of the KSE module with T2A peptide into the strain Lim 11. However, the obtained strains yielded lower titers than the parental one, which might be attributed to the inefficient expression and cleavage of the target proteins mediated by the 2A peptide as previously reported [24]. The results herein suggested that the metabolic flux in the MVA should be systematically manipulated for improved limonene production in the future works.

### Limonene production on flasks with engineered *R. toruloides*

**Fig. 5:**
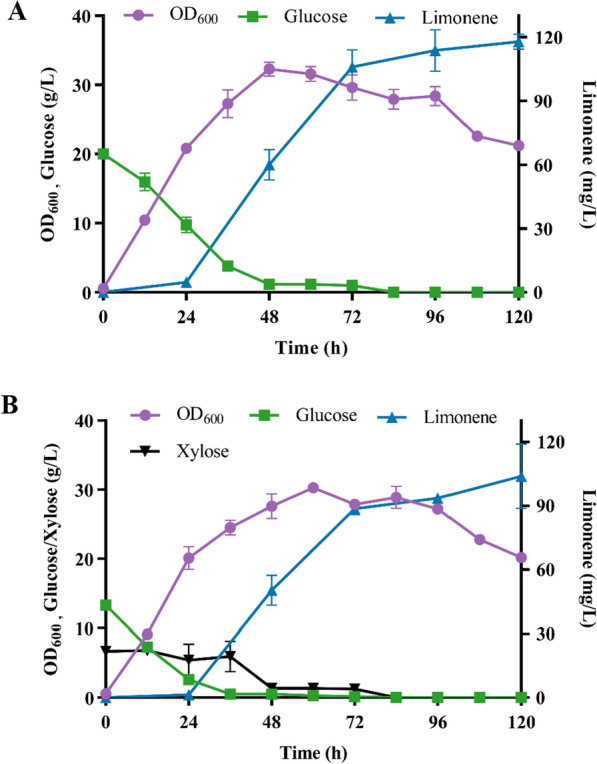
Limonene production in shake flask

To test the potential of the engineered strains for scaling up cultivation, top ten limonene producers in 50-mL test tubes were re-screened twice on 250-mL flasks, and only the strains showed stable cell growth, sugar consumption and limonene titer were utilized for further study (Fig. 5; Additional file 1: Fig S5–S7). Most of the high producers on 50-mL test tubes yielded undesirable titers when cultivated on 250-mL flasks (Fig. 4; Additional file 1: Fig S5). The resultant strain lin24-6 produced 117.8 mg/L limonene within 120 h using glucose while 103.8 mg/L limonene was obtained with the mixture of glucose and xylose as the carbon source (Fig. 5). The obtained titers were much lower than the tolerant limit of the *R. toruloides* (Additional file 1: Fig S8). The cell growth ceased at 72 h while the limonene production stopped at 108 h, 36 h later than the cell growth, suggesting that the yeast *R. toruloides* could recruit the intracellularly reserved sources to support the limonene biosynthesis. Besides, the results implied that the engineered strains can also be deployed to produce limonene by using hydrolysates of lignocellulosic materials. However, the maximum limonene yield was a mere 6 mg/g glucose consumed.

Multiparameters including the carbon and nitrogen sources, aeration, pH, temperature and cultivation volume influence the performance of the engineered strains (Additional file 1: Fig S9) [9]. Owing to its high hydrophobicity and volatility, the dodecane overlay was critical for efficient limonene recovery on 250-mL shake flasks. As is shown in Additional file 1: Fig S6, no limonene was detected when the dodecane overlay was absent. Besides, factors like rotation rate, fermentation volume and dodacane ratio should be further investigated. Although it is possible to elevate the titers and yields via bioprocess optimization [8], in-depth mechanism that hinders limonene production should be elucidated, and extensive metabolic engineering on streamlining the metabolic flux for limonene biosynthesis should be conducted thereafter [3].

## Conclusion

In the present study, we have successfully constructed a limonene biosynthesis pathway in the oleaginous and carotenogenic yeast *R. toruloides* via 2A peptide-mediated multiple protein production*.* The best producers carrying RttHMGR, MfMvaE, MfMvaS, MmKK, and the chimeric SltNPPS1-CltLS1 produced a maximum limonene titer of 383.5 mg/L in 50 mL test tube and 117.8 mg/L in 250 shake flasks. We demonstrated that MfMvaE, MfMvaS, MmKK were efficient in supporting limonene biosynthesis, and the chimeric monoterpene synthase SltNPPS1-CltLS1 harbors higher efficiency in conversion of NPP to limonene in *R. toruloides*. We also found that most of the top-10 producers on 50-mL test tube presented unsatisfactory titers in 250-mL shake flasks. The fermentation conditions should be extensively optimized on 250-mL shake flasks. Our results would also provide insights into microbial production of other valuable monoterpenes with *R. toruloides*.

## Material and methods

### Strains and culture media

*Escherichia coli* DH5α were used for all the routine cloning and plasmid construction. *E. coli* were cultivated at 37 °C, 200 rpm, in 50 mL test tube with 5 mL Luria–Bertani broth (10 g/L tryptone, 5 g/L yeast extract, and 10 g/L NaCl) supplemented with 50 μg/mL kanamycin for plasmid selection.

The wild type and the carotenogenesis-deficient *R. toruloides* NP11 were used as the parent strains, and all the strains used in this study are listed in Additional file 2: Table S1. The *R. toruloides* strains were cultivated in YPD (20 g/L glucose, 20 g/L peptone, and 10 g/L yeast extract) medium at 28 °C, shaking at 200 rpm. The engineered strains were selected and grown on selection medium or plates, which were supplemented with 50 μg/mL antibiotics (Hygromycin B or Nourseothricin or Zeocin). Screening plates for *E. coli* and *R. toruloides* were prepared by adding 20 g/L agar into the corresponding liquid media. Microbial growth medium was purchased from AOBOX (Beijing, China). Molecular biology reagents, enzymes, and kits were from Takara Biomedical Technology Co., Ltd. (Beijing, China), Vazyme Biotech Co., Ltd. (Nanjing, China) and Sangon Biotech Co., Ltd. (Shanghai, China). Nourseothricin sulfate was obtained from Gold Biotechnology (Saint Louis, USA). Hygromycin B was purchased from Roche diagnostics GmbH (Mannheim, Germany). All other chemicals were purchased from Sigma-Aldrich and Macklin (Shanghai, China).

### Plasmid construction

LS used in the study (GenBank AAL17636.1 (*A. rugosa*), AAG31436.1 (*P. frutescens*), AAM53944.1 and AAM53946.1 (*C. limon*), A0A1C9J6A7.2 (*C. sinensis*), ABW86881.1 (*M. piperita*), BAC92722.1 (*C. obtusa*), and RVW66672.1 (*V. vinifera*)). SLNPPS1 (NM_001247704.1)*. EfmvaE* (GenBank: AF290092) and *EfmvaS* (GenBank: AF290092) from *E. faecalis*, and the *Mm*MK (GenBank: AAM31458) from *M. mazei*. IDI from *P. rhodozyma* (O42641.1). All the amino sequences of the enzymes mentioned above are supplied in Additional file 2: Table S2, and their corresponding gene sequences were codon optimized and synthesized by Genewiz Inc. (Suzhou, China). The native HMGR (EMS19753.1) and MPK (XP_016272321.1) from *R. toruloides* (XP_016272321.1) were cloned using the *R. toruloides* NP11 cDNA as the template and overexpressed with 798 amino acids of the N-terminal transmembrane domain truncated. The sequences of P2A (porcine teschovirus-1), T2A (Thosea asigna virus) and F2A (foot-and-mouth disease virus) peptides were synthesized in Synbio Tech (Suzhou China) [22]. The resulting fragments were inserted into plasmid pZPK.

All the vectors were constructed based on the p*ZPK*-P_*PGK*_*-*Hyg-T_*NOS*_-P_*GPD*_-MCS-T_*HSP*_ plasmid [33], which could be selected in both *E. coli* and *Agrobacterium tumefaciens* with kanamycin. The primers for cloning target genes are shown in Additional file 2: Table S3, and the obtained PCR fragments and the vectors were digested with *Eco*R V/*Spe* I, respectively, and then ligated using the DNA ligation kit (Takara). The resulted vectors are listed in Additional file 2: Table S4.

### ATMT procedure and screening of recombinant *R. toruloides*

The correct vectors were transformed into competent *A. tumefaciens* AGL1, and the ATMT experiment was conducted as previously reported [33]. First, the vector carrying *A. tumefaciens* cells were cultivated in LB medium supplemented with 50 μg/mL kanamycin at 28 ℃ for 15 h, and *R. toruloides* NP11 cells were cultured in YPD medium at 30 ℃ for 15 h. Second, cells were collected by centrifugation at 15,000*g* for 30 s, then washed and diluted with sterilized water to an optical density at 600 nm (OD_600_) of 0.6. For co-culture, 100 μL of each cell suspension was mixed, spread onto the IM agar plates containing 200 μM acetosyringone, and incubated at 25 ℃ for 2 days. Last, the transformant was transferred onto the selection plate to incubate at 28 ℃ for 2 days. Then the transformants were streaked on selection YPD plates at 28 ℃ for five successive generations, and only those with stable phenotypic traits were collected for further characterization.

### Fermentation

For primary screening of the limonene producing strains, 50-mL test tube was used with a loading volume of 5 mL. Briefly, single colonies were inoculated into 50-mL tubes containing 5 mL selection YPD liquid medium, and maintained at 28 ℃, 200 rpm for 120 h. For facilitating limonene extraction and alleviation of its cytotoxicity to host strains, 20% (v/v) *n*-dodecane was added.

For flask fermentation, the obtained strains were precultured in 250-mL shaking flasks, containing 50 mL YPD at 28 ℃, 200 rpm for 36 h until the *OD*_*600*_ reached 10–12. Then, the precultures were inoculated into 250-mL shake flasks (containing 50 mL YPD) with an initial cell density of *OD*_*600*_ = 1.0, and cultivated at 28 ℃ and 200 rpm. The carbon sources used for limonene production were either 20 g/L glucose or a mixture of glucose and xylose (w/w = 2/1). A cover layer *n*-dodecane comprising 20% of the culture volume was added. Samples and standards were transferred to the gas chromatograph (GC) for qualitative analysis and quantification.

### Homology modeling and structural analysis by computational simulation

Since the structures of the fusion proteins (CltLS-SltNPPS and SltNPPS-CltLS) have not been characterized, we submitted their amino acid sequences to web-based tool I-TASSER for structure predicting (http://zhanglab.ccmb.med.umich.edu/I-TASSER/) [40]. To select the models, I-TASSER uses the SPICKER program to cluster all the decoys based on the pair-wise structure similarity, and reports five models which corresponds to the five largest structure clusters. The models were visualized using versatile molecule model rendering software, PyMOL Version 2.5, and the final models of fusion proteins were selected by aligning to the protein models of CltLS1 and SltNPPS1 which were also predicted using I-TASSER. The proteins transit peptides of LS and NPPS were removed according to previous report [10].

### Extraction and quantification of limonene

For quantification of limonene, 1 mL of *n*-hexane was added to 6 mL of the two-phase culture sample. The samples were vortexed for 2 min then centrifuged at 12,000×*g* for 5 min. Prior to gas chromatography (GC) analysis, the organic phase was dried over anhydrous Na_2_SO_4_. GC analyses were carried out on a Shimadzu GC-2014C GC (Shimadzu, Japan), equipped with a KB-1 column (60 m × 0.25 mm × 0.25 µm, Kromat, USA) and a flame ionization detector. Nitrogen was used as the carrier gas at 1.0 mL/min constant flow rate and the injection volume was set 1 µL. The GC oven temperature was 145 °C held for 27 min. The injector was maintained at 240 °C and the detector temperature was 260 °C. The split ratio was 20:1. Limonene quantification was computed according to the (R)-limonene standard curve (Sigma, USA).

## Supplementary Information

**Additional file 1: Fig S1**. Limonene producing gene modules. **Fig S2**. Monoterpene profile of engineered *Rhodosporidium toruloides* Np11. **Fig S3**. Characterization of the Limonene Configuration. **Fig S4**. The limonene synthases utilized in *R. toruloides*. **Fig S5**. Effect of fermentation system on limonene production. **Fig S6**. Effects of dodecane overlay on limonene production with engineered *R. toruloides* strains. **Fig S7**. Comparison of limonene production of engineered *R. toruloides* strains in shake flasks. **Fig S8**. Limonene tolerance of *R. toruloides*. **Fig S9**. The influence of working volume on limonene production in shake flasks.

**Additional file 2: Table S1**. Strains genotype and limonene titers. **Table S2**. Protein sequence and source in this study. **Table S3**. Primer sequence used in vector preparation. **Table S4**. Plasmids used in this study.

## Data Availability

The datasets used and/or analyzed during the current study are available from the corresponding author on reasonable request.
