# Supplementary material for: Engineering Rhodosporidium toruloides for limonene production
Source: Biotechnol Biofuels. 2021 Dec 22;14:243. doi: 10.1186/s13068-021-02094-7 (PMC8697501; doi:10.1186/s13068-021-02094-7)
Supplement: Supplementary file 1 — Additional file 1: Fig S1. Limonene producing gene modules. Fig S2. Monoterpene profile of engineered Rhodosporidium toruloides Np11. Fig S3. Characterization of the Limonene Configuration. Fig S4. The limonene synthases utilized in R. toruloides. Fig S5. Effect of fermentation system on limonene production. Fig S6. Effects of dodecane overlay on limonene production with engineered R. toruloides strains. Fig S7. Comparison of limonene production of engineered R. toruloides strains in shake flasks. Fig S8. Limonene tolerance of R. toruloides. Fig S9. The influence of working volume on limonene production in shake flasks. [file 13068_2021_2094_MOESM1_ESM.pptx]

## Slide 1
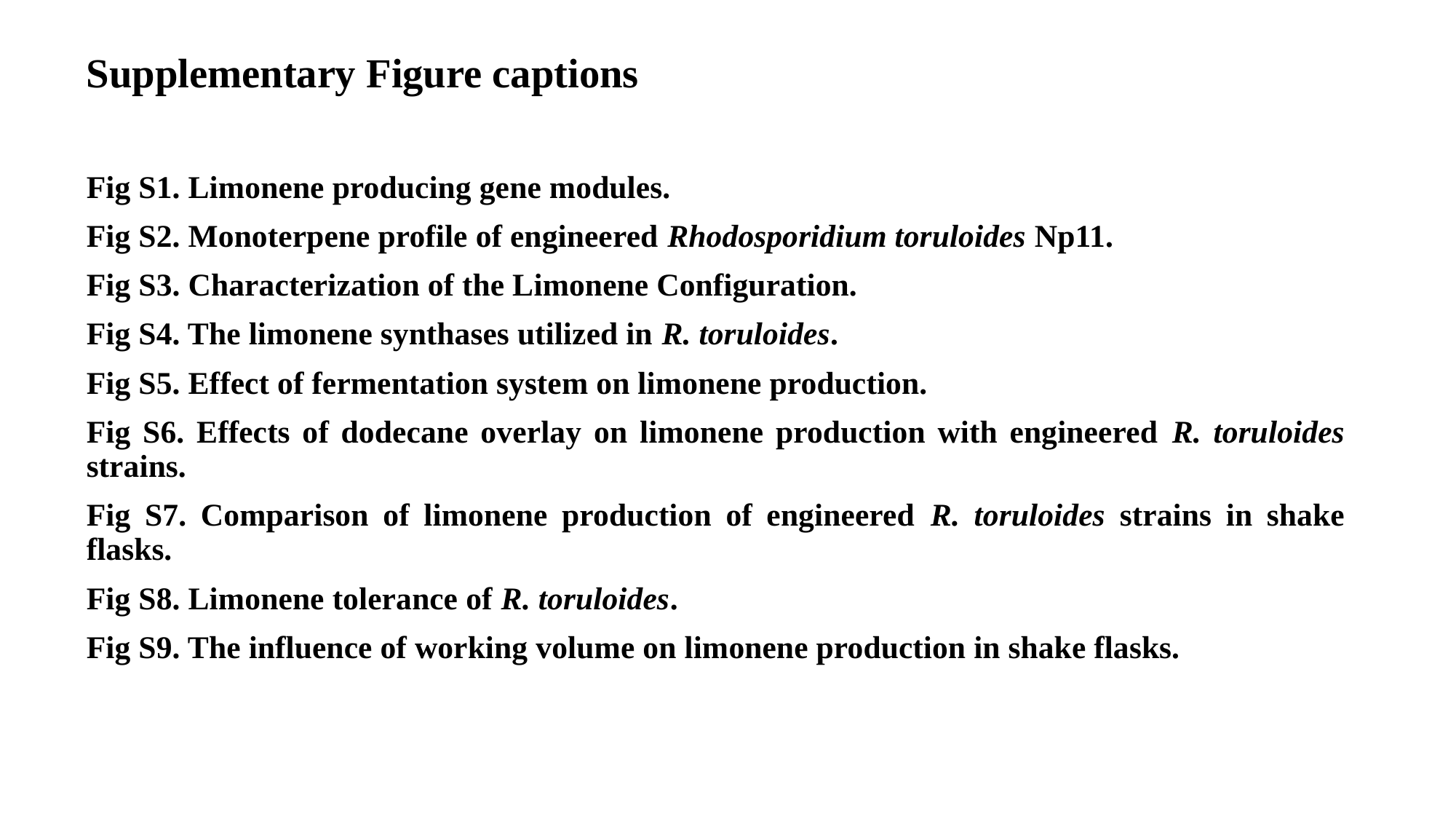

Supplementary Figure captions
Fig S1. Limonene producing gene modules.
Fig S2. Monoterpene profile of engineered Rhodosporidium toruloides Np11.
Fig S3. Characterization of the Limonene Configuration.
Fig S4. The limonene synthases utilized in R. toruloides.
Fig S5. Effect of fermentation system on limonene production.
Fig S6. Effects of dodecane overlay on limonene production with engineered R. toruloides strains.
Fig S7. Comparison of limonene production of engineered R. toruloides strains in shake flasks.
Fig S8. Limonene tolerance of R. toruloides.
Fig S9. The influence of working volume on limonene production in shake flasks.

## Slide 2
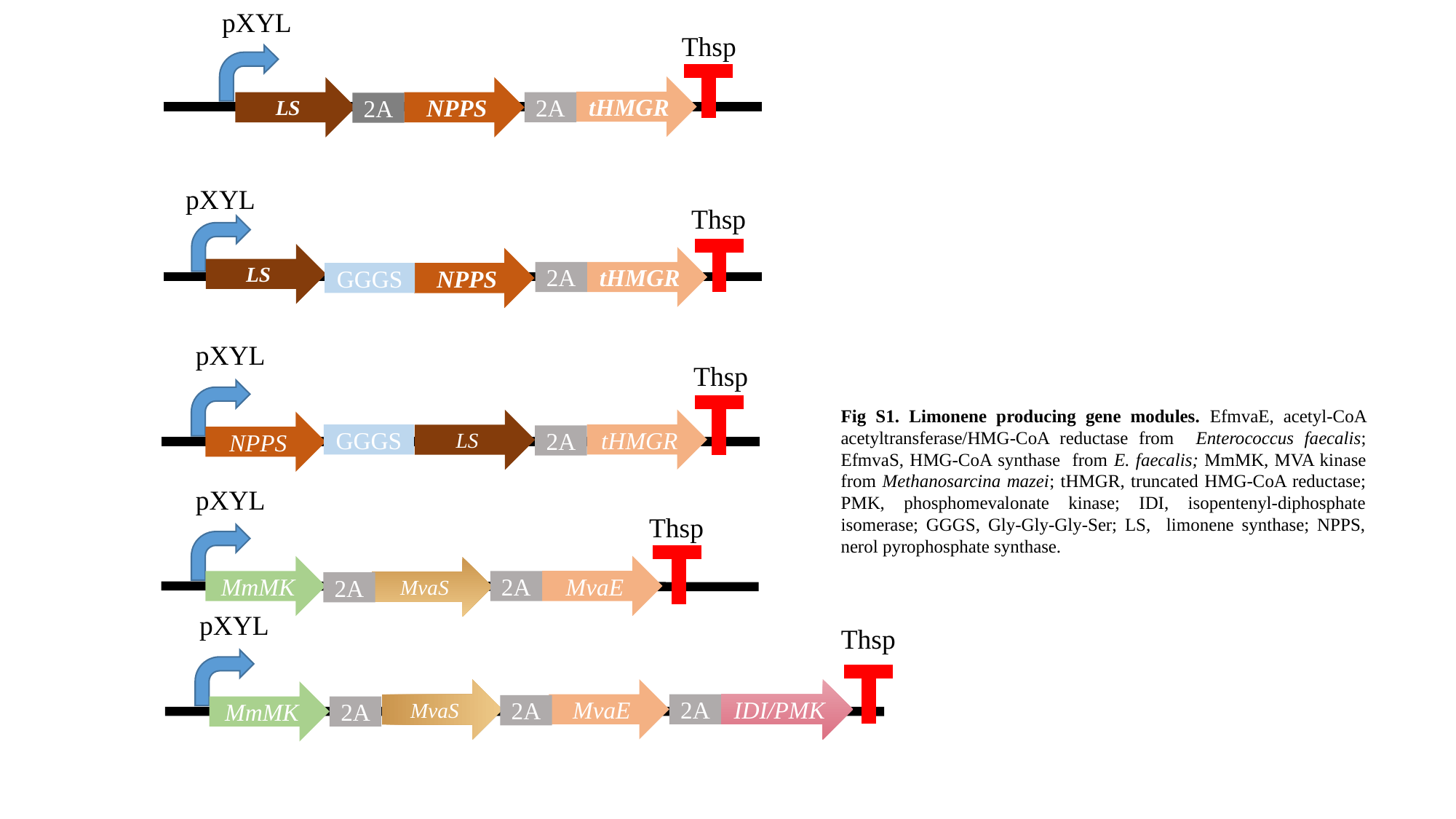

pXYL
Thsp
tHMGR
LS
NPPS
2A
2A
pXYL
Thsp
LS
tHMGR
NPPS
2A
GGGS
pXYL
Thsp
tHMGR
LS
NPPS
GGGS
2A
Fig S1. Limonene producing gene modules. EfmvaE, acetyl-CoA acetyltransferase/HMG-CoA reductase from Enterococcus faecalis; EfmvaS, HMG-CoA synthase from E. faecalis; MmMK, MVA kinase from Methanosarcina mazei; tHMGR, truncated HMG-CoA reductase; PMK, phosphomevalonate kinase; IDI, isopentenyl-diphosphate isomerase; GGGS, Gly-Gly-Gly-Ser; LS, limonene synthase; NPPS, nerol pyrophosphate synthase.
pXYL
Thsp
MvaE
MmMK
MvaS
2A
2A
pXYL
Thsp
MvaE
MvaS
MmMK
2A
IDI/PMK
2A
2A

## Slide 3
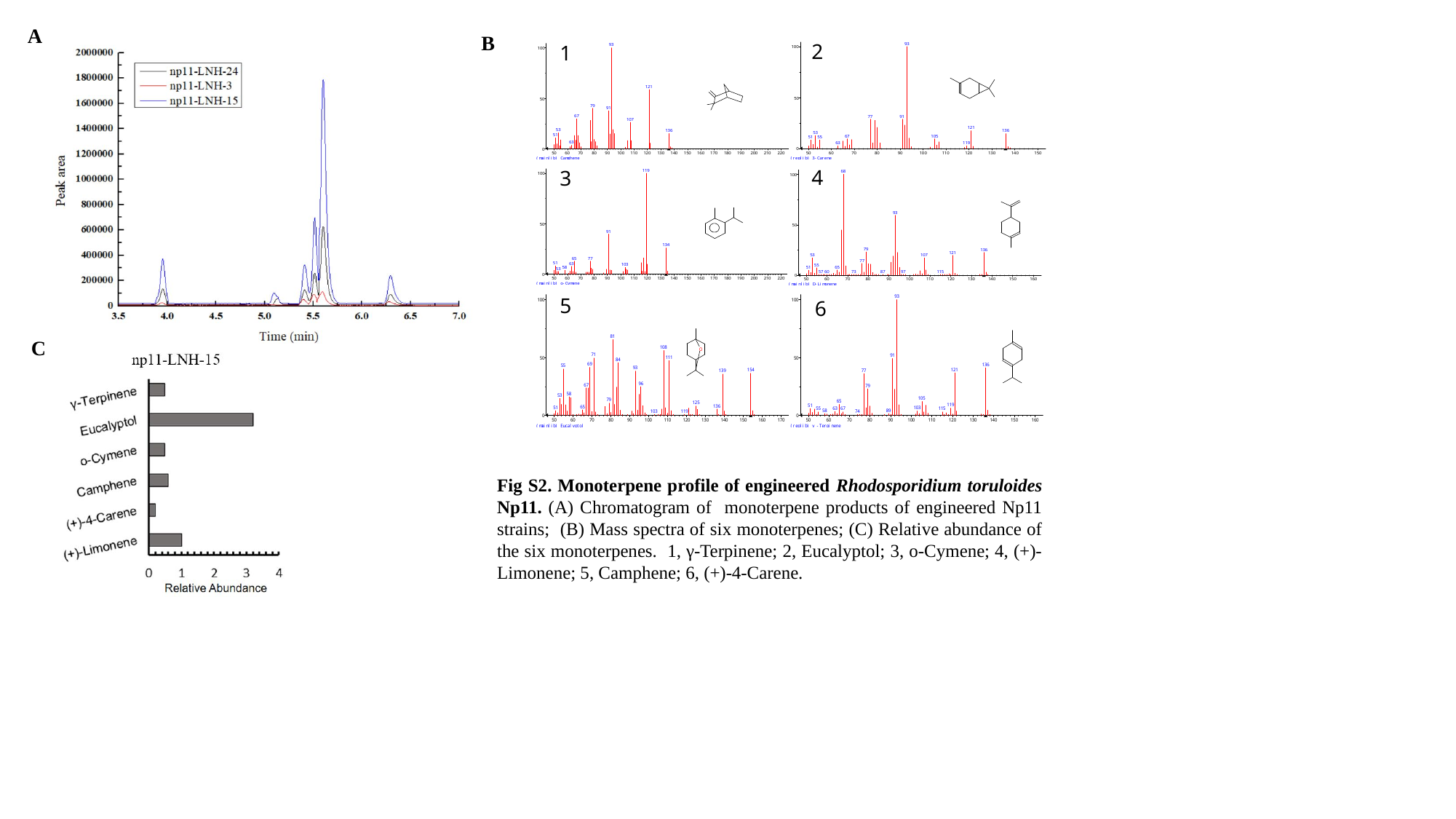

A
B
2
1
3
4
5
6
C
Fig S2. Monoterpene profile of engineered Rhodosporidium toruloides Np11. (A) Chromatogram of monoterpene products of engineered Np11 strains; (B) Mass spectra of six monoterpenes; (C) Relative abundance of the six monoterpenes. 1, γ-Terpinene; 2, Eucalyptol; 3, o-Cymene; 4, (+)-Limonene; 5, Camphene; 6, (+)-4-Carene.

## Slide 4
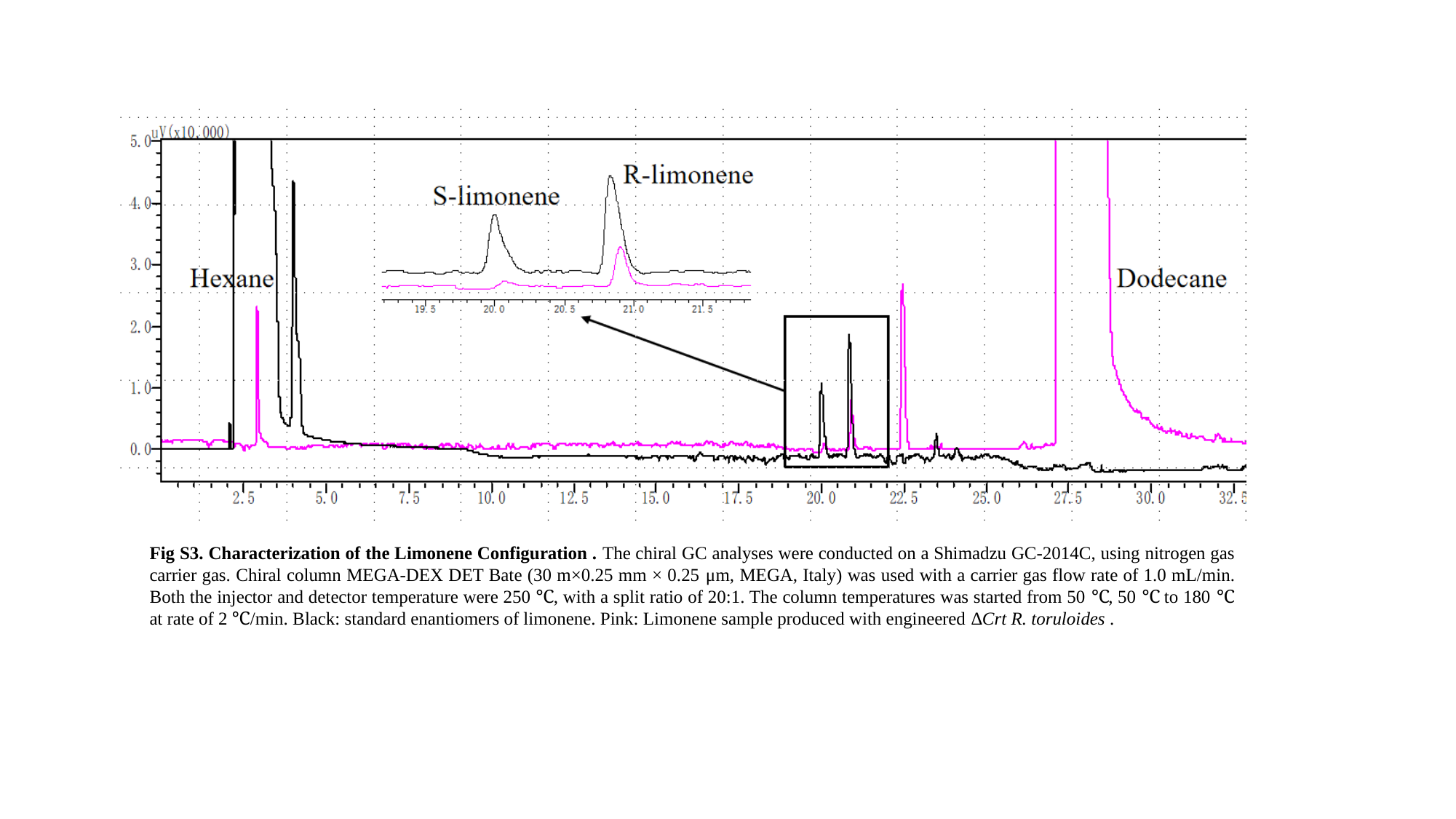

Fig S3. Characterization of the Limonene Configuration . The chiral GC analyses were conducted on a Shimadzu GC-2014C, using nitrogen gas carrier gas. Chiral column MEGA-DEX DET Bate (30 m×0.25 mm × 0.25 μm, MEGA, Italy) was used with a carrier gas flow rate of 1.0 mL/min. Both the injector and detector temperature were 250 ℃, with a split ratio of 20:1. The column temperatures was started from 50 ℃, 50 ℃ to 180 ℃ at rate of 2 ℃/min. Black: standard enantiomers of limonene. Pink: Limonene sample produced with engineered ∆Crt R. toruloides .

## Slide 5
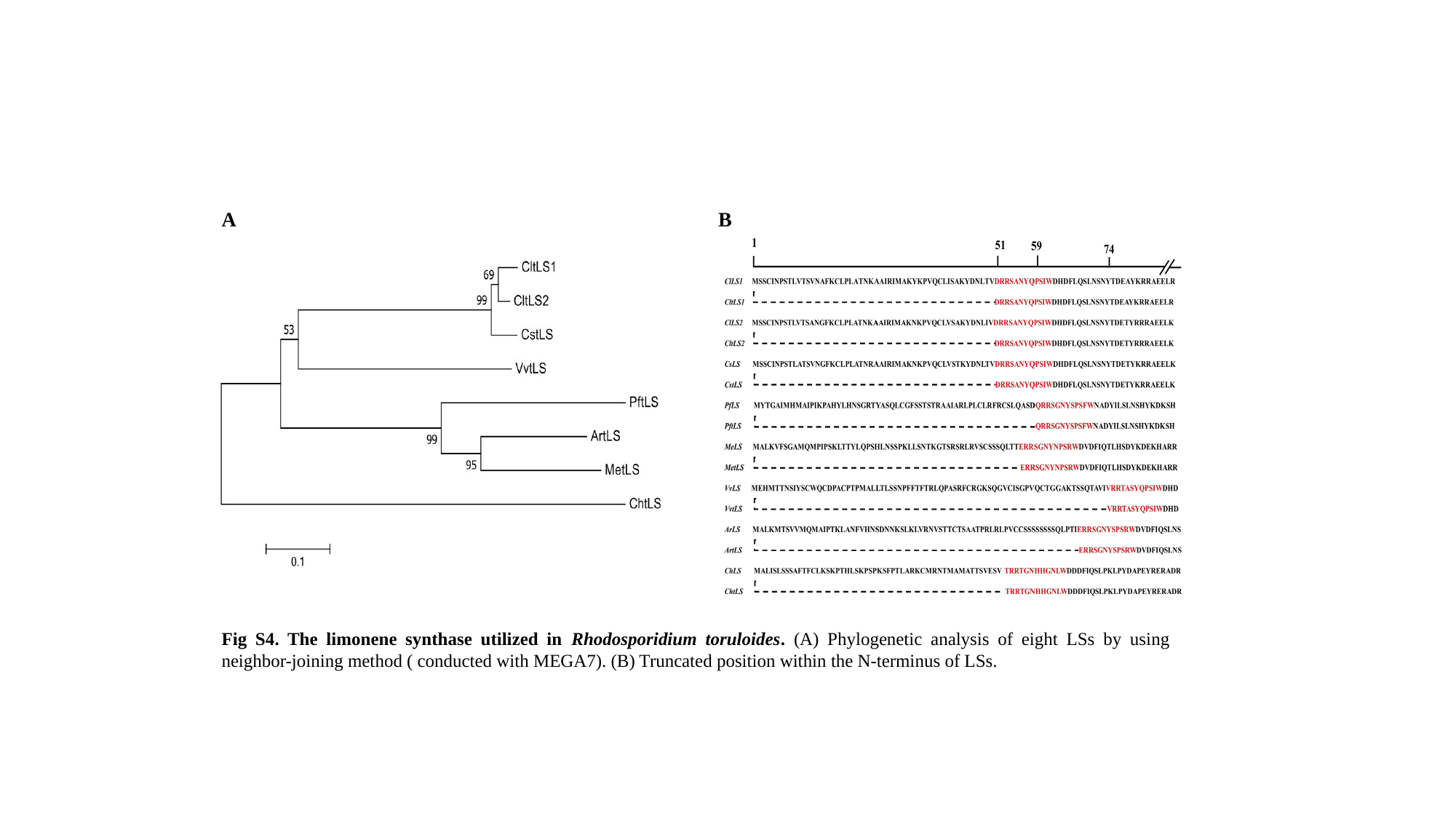

A
B
Fig S4. The limonene synthase utilized in Rhodosporidium toruloides. (A) Phylogenetic analysis of eight LSs by using neighbor-joining method ( conducted with MEGA7). (B) Truncated position within the N-terminus of LSs.

## Slide 6
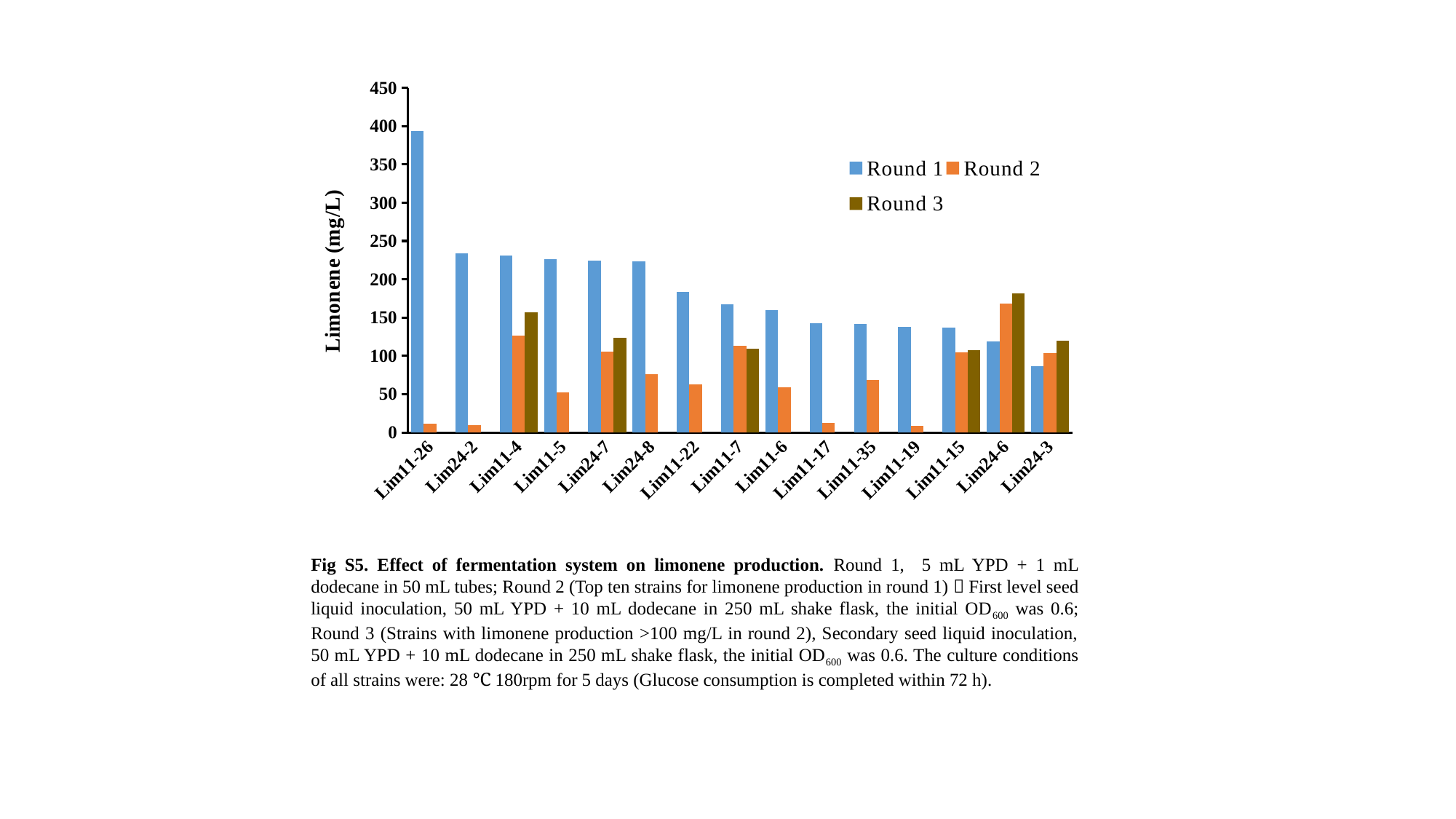

### Chart
| Category | Round 1 | Round 2 | Round 3 |
|---|---|---|---|
| Lim11-26 | 393.48021662098 | 11.1733968882094 | None |
| Lim24-2 | 234.307120411847 | 9.68027700332764 | None |
| Lim11-4 | 230.586053352945 | 126.129148304704 | 156.610256184135 |
| Lim11-5 | 225.878424817811 | 52.1552297868513 | None |
| Lim24-7 | 224.835702346727 | 105.997841532512 | 123.461792848009 |
| Lim24-8 | 223.33887811727 | 76.2599154600234 | None |
| Lim11-22 | 183.147930734773 | 62.7488982822196 | None |
| Lim11-7 | 167.294216754697 | 113.354977965644 | 109.114775783765 |
| Lim11-6 | 159.882837467407 | 59.2130587283029 | None |
| Lim11-17 | 142.792779300662 | 12.6896303624427 | None |
| Lim11-35 | 141.663141004212 | 68.475042719669 | None |
| Lim11-19 | 138.248285083907 | 9.051803219714 | None |
| Lim11-15 | 137.284722872234 | 104.835506790179 | 107.67891000485 |
| Lim24-6 | 118.552356756034 | 168.185088587103 | 181.229860223114 |
| Lim24-3 | 86.3520492077288 | 103.313697274935 | 119.757484897923 |Fig S5. Effect of fermentation system on limonene production. Round 1, 5 mL YPD + 1 mL dodecane in 50 mL tubes; Round 2 (Top ten strains for limonene production in round 1)，First level seed liquid inoculation, 50 mL YPD + 10 mL dodecane in 250 mL shake flask, the initial OD600 was 0.6; Round 3 (Strains with limonene production >100 mg/L in round 2), Secondary seed liquid inoculation, 50 mL YPD + 10 mL dodecane in 250 mL shake flask, the initial OD600 was 0.6. The culture conditions of all strains were: 28 ℃ 180rpm for 5 days (Glucose consumption is completed within 72 h).

## Slide 7
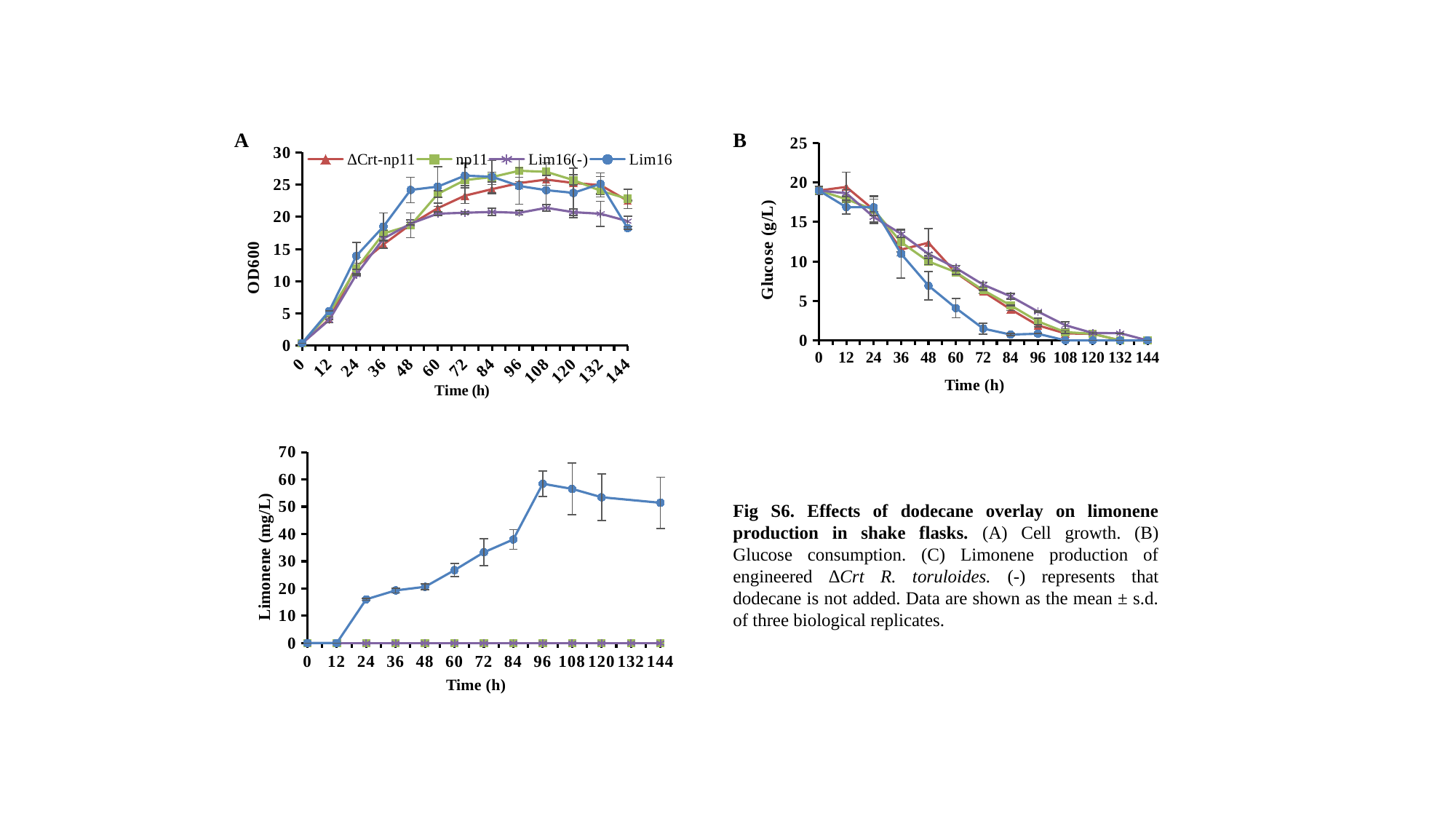

A
B
### Chart
| Category | ΔCrt-np11 | np11 | Lim16(-) | Lim16 |
|---|---|---|---|---|
| 0 | 18.983650225039 | 18.983650225039 | 18.983650225039 | 18.983650225039 |
| 12 | 19.4396987232479 | 17.8873886286397 | 18.6313952420318 | 16.8770092771195 |
| 24 | 16.5463396711674 | 16.5555249379995 | 15.6278129879673 | 16.8678240102875 |
| 36 | 11.5036281803986 | 12.4405253972628 | 13.506016349775 | 10.9708827041426 |
| 48 | 12.3684210526316 | 9.9986222099752 | 10.9433269036466 | 6.93303940479471 |
| 60 | 8.56709837420777 | 8.64517314227978 | 9.16873335170387 | 4.08928079360705 |
| 72 | 6.17341783778819 | 6.35712317442822 | 7.06071461375953 | 1.49352438688344 |
| 84 | 3.93827500688895 | 4.43979057591623 | 5.57867181041609 | 0.739459906310278 |
| 96 | 1.8995131808579 | 2.41044364838799 | 3.64976577569578 | 0.842013410489575 |
| 108 | 0.886561954624782 | 1.04225222742721 | 1.92247634793791 | 0.0 |
| 120 | 0.829613300266373 | 0.842931937172775 | 0.926839349683108 | 0.0 |
| 132 | 0.0 | 0.0 | 0.915954808487187 | 0.0 |
| 144 | 0.0 | 0.0 | 0.0 | 0.0 |
### Chart
| Category | ΔCrt-np11 | np11 | Lim16(-) | Lim16 |
|---|---|---|---|---|
| 0 | 0.370666666666667 | 0.393666666666667 | 0.384333333333333 | 0.397 |
| 12 | 4.11533333333333 | 5.022 | 3.98933333333333 | 5.388 |
| 24 | 12.2686666666667 | 11.977 | 11.06 | 13.9493333333333 |
| 36 | 15.7273333333333 | 17.4116666666667 | 16.6263333333333 | 18.507 |
| 48 | 18.8376666666667 | 18.6826666666667 | 18.9556666666667 | 24.1763333333333 |
| 60 | 21.3473333333333 | 23.5613333333333 | 20.5 | 24.6956666666667 |
| 72 | 23.288 | 25.707 | 20.6366666666667 | 26.404 |
| 84 | 24.2993333333333 | 26.1716666666667 | 20.7733333333333 | 26.2263333333333 |
| 96 | 25.2423333333333 | 27.1556666666667 | 20.623 | 24.7913333333333 |
| 108 | 25.789 | 26.9916666666667 | 21.402 | 24.149 |
| 120 | 25.2696666666667 | 25.707 | 20.7323333333333 | 23.7253333333333 |
| 132 | 24.928 | 24.0533333333333 | 20.4863333333333 | 25.133 |
| 144 | 22.4953333333333 | 22.796 | 19.3383333333333 | 18.2586666666667 |
### Chart
| Category | ΔCrt-np11 | np11 | Lim16(-) | Lim16 |
|---|---|---|---|---|
| 0 | 0.0 | 0.0 | 0.0 | 0.0 |
| 12 | 0.0 | 0.0 | 0.0 | 0.0 |
| 24 | 0.0 | 0.0 | 0.0 | 16.0383664250897 |
| 36 | 0.0 | 0.0 | 0.0 | 19.2989407779064 |
| 48 | 0.0 | 0.0 | 0.0 | 20.6298154580671 |
| 60 | 0.0 | 0.0 | 0.0 | 26.7354517775205 |
| 72 | 0.0 | 0.0 | 0.0 | 33.2724387838243 |
| 84 | 0.0 | 0.0 | 0.0 | 37.9933959432223 |
| 96 | 0.0 | 0.0 | 0.0 | 58.4161127549781 |
| 108 | 0.0 | 0.0 | 0.0 | 56.5317552210644 |
| 120 | 0.0 | 0.0 | 0.0 | 53.4675300541761 |
| 132 | 0.0 | 0.0 | 0.0 | None |
| 144 | 0.0 | 0.0 | 0.0 | 51.4433151793244 |Fig S6. Effects of dodecane overlay on limonene production in shake flasks. (A) Cell growth. (B) Glucose consumption. (C) Limonene production of engineered ∆Crt R. toruloides. (-) represents that dodecane is not added. Data are shown as the mean ± s.d. of three biological replicates.

## Slide 8
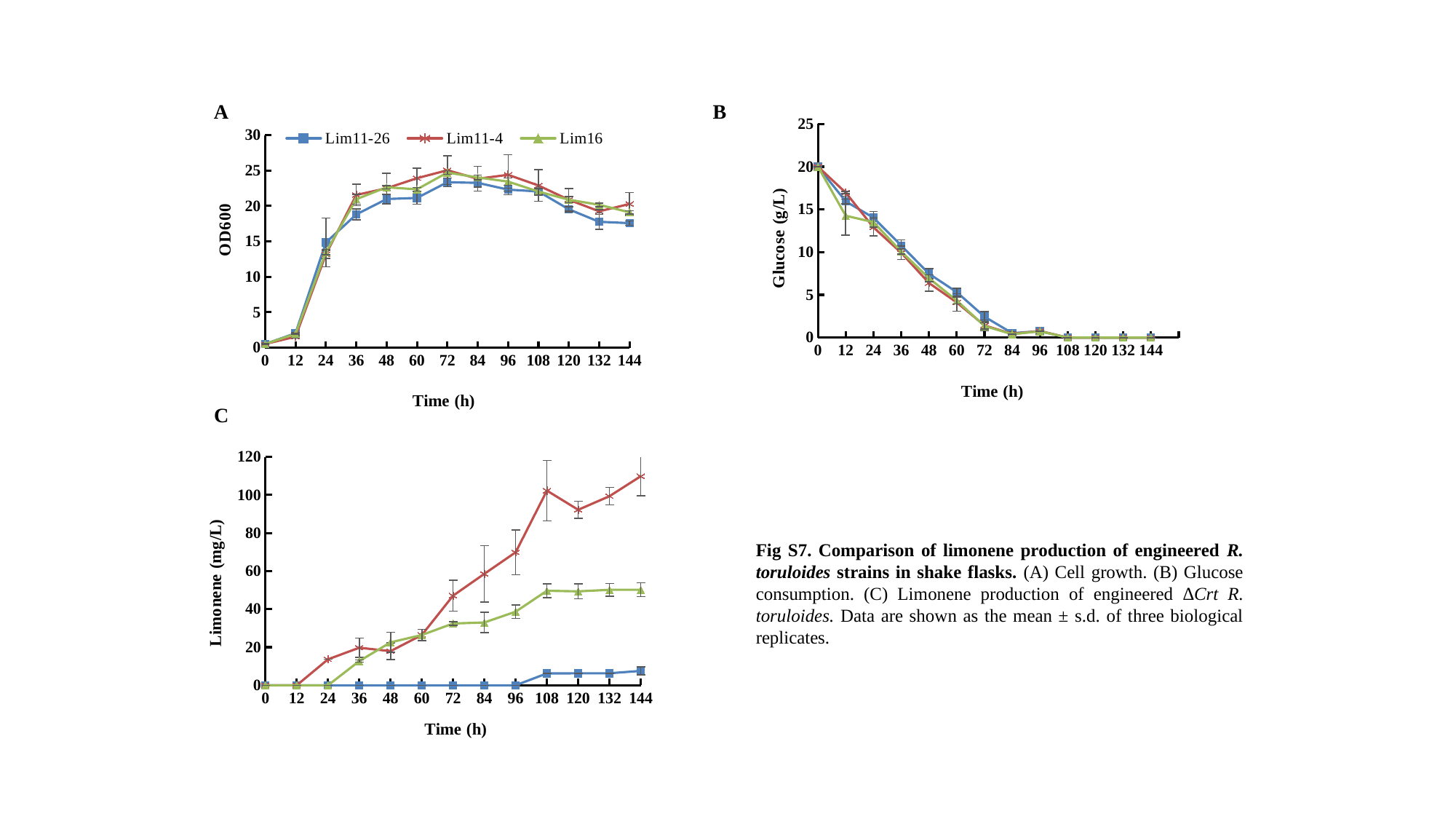

B
A
### Chart
| Category | Lim11-26 | Lim11-4 | Lim16 |
|---|---|---|---|
| 0 | 0.527333333333333 | 0.527333333333333 | 0.527333333333333 |
| 12 | 2.043 | 1.5516 | 1.956 |
| 24 | 14.8413333333333 | 13.167 | 13.482 |
| 36 | 18.8053333333333 | 21.5523333333333 | 20.951 |
| 48 | 20.9783333333333 | 22.4953333333333 | 22.632 |
| 60 | 21.1286666666667 | 23.9166666666667 | 22.345 |
| 72 | 23.3426666666667 | 25.0373333333333 | 24.7093333333333 |
| 84 | 23.2606666666667 | 23.8346666666667 | 24.026 |
| 96 | 22.304 | 24.395 | 23.4383333333333 |
| 108 | 22.0443333333333 | 22.8916666666667 | 22.0306666666667 |
| 120 | 19.5296666666667 | 20.869 | 20.8826666666667 |
| 132 | 17.7666666666667 | 19.2426666666667 | 20.1583333333333 |
| 144 | 17.5753333333333 | 20.2813333333333 | 19.106 |
[unsupported chart]
C
### Chart
| Category | Lim11-26 | Lim11-4 | Lim16 |
|---|---|---|---|
| 0 | 0.0 | 0.0 | 0.0 |
| 12 | 0.0 | 0.0 | 0.0 |
| 24 | 0.0 | 13.6387283236994 | 0.0 |
| 36 | 0.0 | 19.7237900940723 | 12.7054856624731 |
| 48 | 0.0 | 17.927235634138 | 22.5409724583475 |
| 60 | 0.0 | 26.4125580868185 | 26.4125580868185 |
| 72 | 0.0 | 47.0839850391023 | 32.451377082625 |
| 84 | 0.0 | 58.5667573387737 | 32.985549132948 |
| 96 | 0.0 | 69.8839963731157 | 38.6657032755299 |
| 108 | 6.24591975518531 | 102.285192111527 | 49.6626430919188 |
| 120 | 6.2665193244928 | 92.2107843137255 | 49.3327382976312 |
| 132 | 6.31355547999547 | 99.3776776606596 | 50.148787260569 |
| 144 | 7.5588518644452 | 109.843930635838 | 50.1912048056217 |Fig S7. Comparison of limonene production of engineered R. toruloides strains in shake flasks. (A) Cell growth. (B) Glucose consumption. (C) Limonene production of engineered ∆Crt R. toruloides. Data are shown as the mean ± s.d. of three biological replicates.

## Slide 9
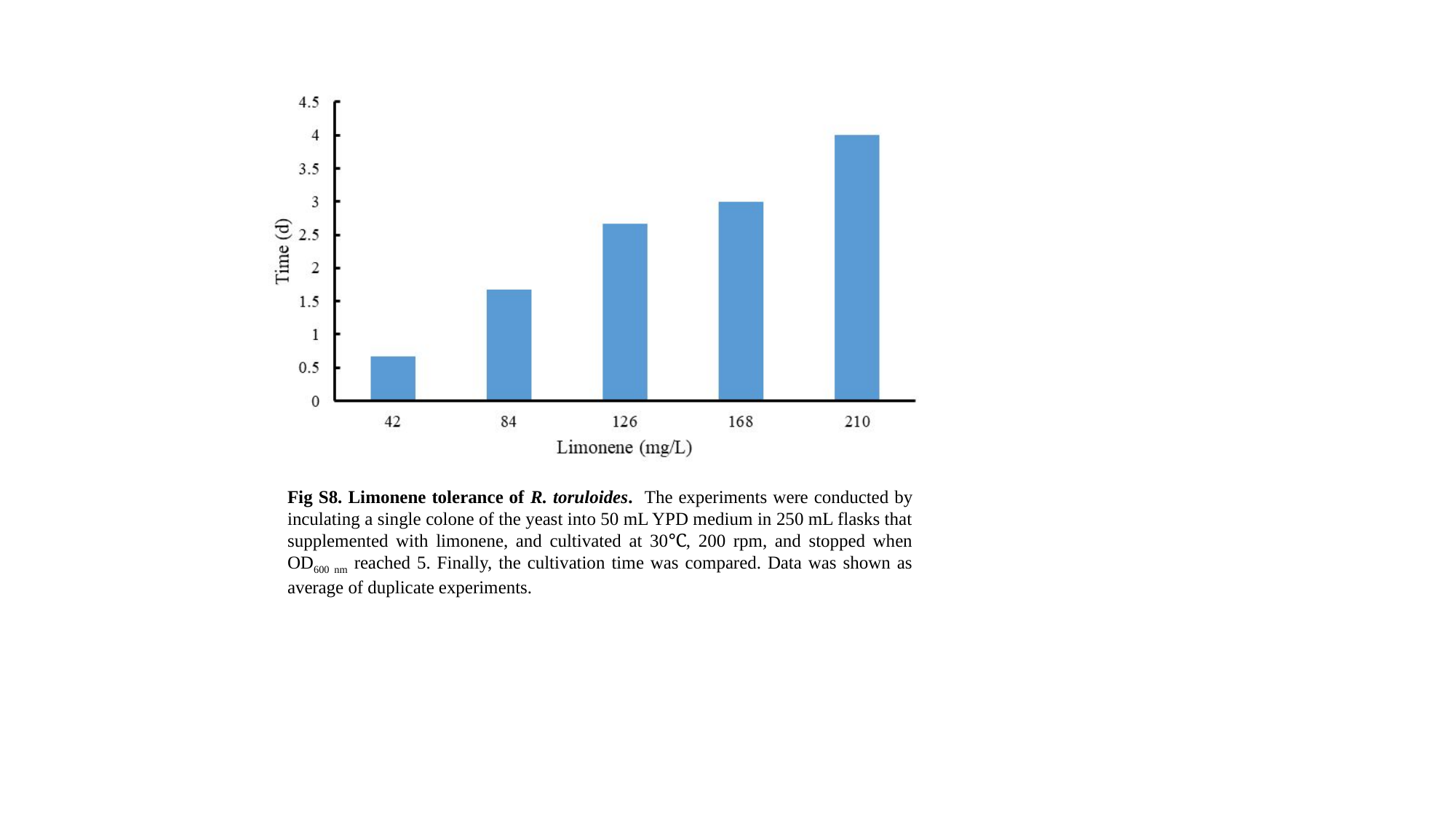

Fig S8. Limonene tolerance of R. toruloides. The experiments were conducted by inculating a single colone of the yeast into 50 mL YPD medium in 250 mL flasks that supplemented with limonene, and cultivated at 30℃, 200 rpm, and stopped when OD600 nm reached 5. Finally, the cultivation time was compared. Data was shown as average of duplicate experiments.

## Slide 10
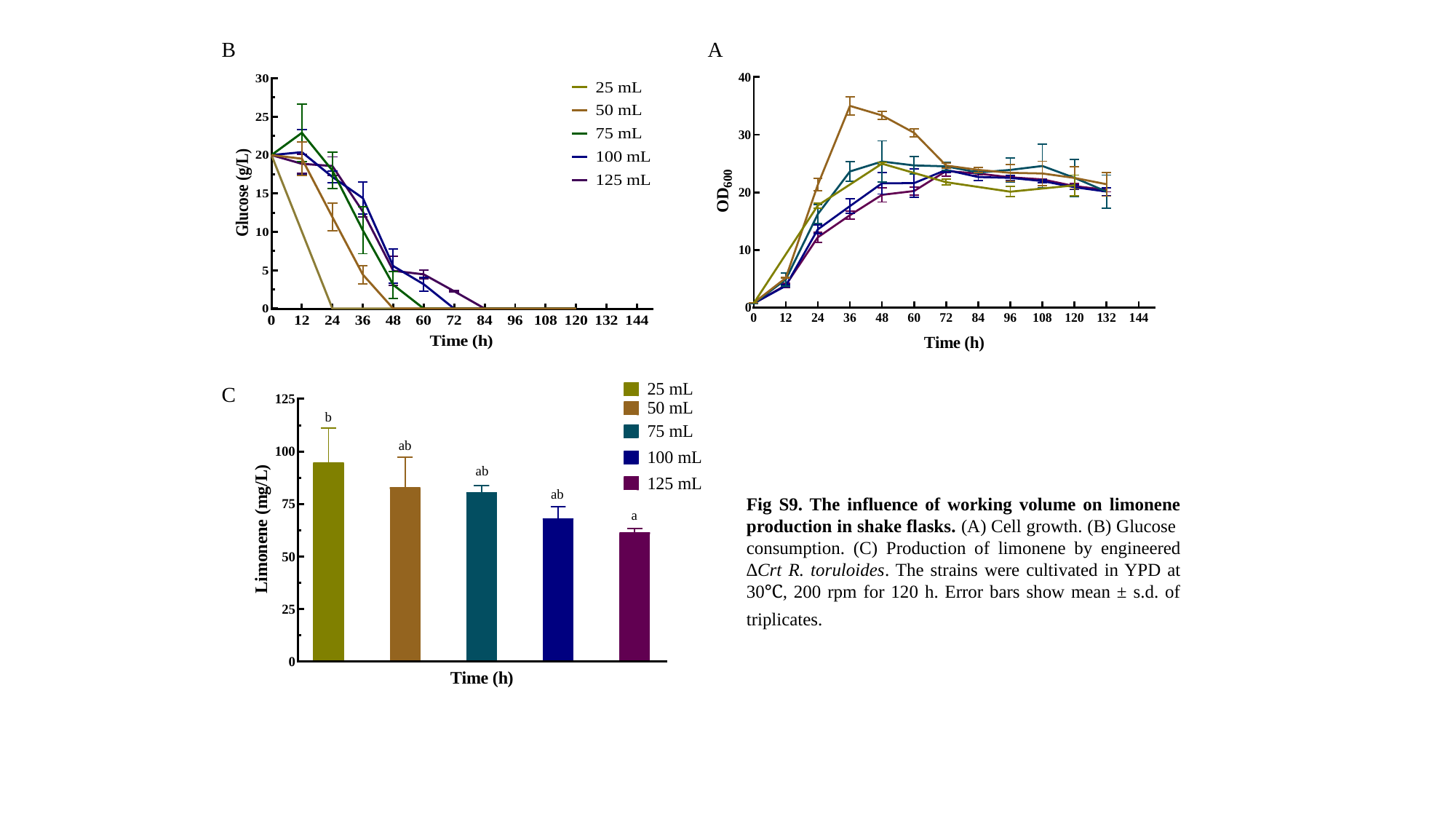

B
A
C
Fig S9. The influence of working volume on limonene production in shake flasks. (A) Cell growth. (B) Glucose consumption. (C) Production of limonene by engineered ∆Crt R. toruloides. The strains were cultivated in YPD at 30℃, 200 rpm for 120 h. Error bars show mean ± s.d. of triplicates.
