# Supplementary material for: Engineering Rhodosporidium toruloides for limonene production
Source: Biotechnol Biofuels. 2021 Dec 22;14:243. doi: 10.1186/s13068-021-02094-7 (PMC8697501; doi:10.1186/s13068-021-02094-7)
Supplement: Supplementary file 2 — Additional file 2: Table S1. Strains genotype and limonene titers. Table S2. Protein sequence and source in this study. Table S3. Primer sequence used in vector preparation. Table S4. Plasmids used in this study. [file 13068_2021_2094_MOESM2_ESM.docx]

Table S1. Strains genotype and limonene titers.

| **In –house name** | **Strains** | **Genotype or characteristic** | **Carbon** | **Average titer(mg/L)** | **Middle titer (mg/L)** | **Max titer (mg/L)** |
| --- | --- | --- | --- | --- | --- | --- |
| Np11 | *R. toruloides* np11 | *MAT A1* | Glucose | 0 | 0 | 0 |
| 4# | *R. toruloides* Y4 | Diploid | Glucose | 0 | 0 | 0 |
| ΔCrt-np11 | *R. toruloides* ΔCrt-np11 | *MAT A1 ΔCrt,* Hyg^R^ | Glucose | 0 | 0 | 0 |
| pGPD-CltLS1-np11 | Pzpk-pGPD-CltLS1-Hyg-np11 | *MAT A1,* p*GPD-CltLS1-Hyg-*Thsp, Hyg^R^ | Glucose | 0 | 0 | 0 |
| pGPD-LN-np11 | Pzpk-pGPD-LN-Ntc-np11 | *MAT A1,* p*GPD-CltLS1-NPPS-Ntc-*Thsp, Ntc^R^ | Glucose | 0.93 | 0 | 5.21 |
| pGPD-LNH-np11 | Pzpk-pGPD-20190821-hyg-np11 | *MAT A1,* p*GPD-CltLS1-NPPS-HMGR-Hyg-*Thsp, Hyg^R^ | Glucose | 0.18 | 0.04 | 1.03 |
| pGPD-LNH-4# | Pzpk-pGPD-20190821-hyg-4# | Diploid, p*GPD-CltLS1-NPPS-HMGR-Hyg-*Thsp, Hyg^R^ | Glucose | 0.13 | 0.10 | 0.88 |
| pAra-LNH-np11 | Pzpk-pAra-2019082-hyg-np11 | *MAT A1,* p*ARA-CltLS1-NPPS-HMGR-Hyg-*Thsp, Hyg^R^ | Arabinose | 0.10 | 0.09 | 0.85 |
| pAra-LNH-np11 | Pzpk-pAra-20190821-hyg-np11 | *MAT A1,* p*ARA-CltLS1-NPPS-HMGR-Hyg-*Thsp, Hyg^R^ | Glucose | 0.04 | 0.00 | 0.59 |
| pAra-LNH-4# | Pzpk-pAra-20190821-hyg-4# | Diploid, p*ARA-CltLS1-NPPS-HMGR-Hyg-*Thsp, Hyg^R^ | Arabinose | 0.09 | 0.06 | 0.48 |
| pAra-LNH-4# | Pzpk-pAra-20190821-hyg-4# | Diploid, p*ARA-CltLS1-NPPS-HMGR-Hyg-*Thsp, Hyg^R^ | Glucose | 0.60 | 0.49 | 1.46 |
| pXyl-LNH-np11 | Pzpk-pXyl-20190821-hyg-np11 | *MAT A1,* p*XYL-CltLS1-NPPS-HMGR-Hyg-*Thsp, Hyg^R^ | Xylose | 0.23 | 0.18 | 0.73 |
| pXyl-LNH-np11 | Pzpk-pXyl-20190821-hyg-np11 | *MAT A1,* p*XYL-CltLS1-NPPS-HMGR-Hyg-*Thsp, Hyg^R^ | Glucose | 0.40 | 0.15 | 6.64 |
| pXyl-LNH-4# | Pzpk-pXyl-20190821-hyg-4# | Diploid, p*XYL-CltLS1-NPPS-HMGR-Hyg-*Thsp, Hyg^R^ | Xylose | 0.17 | 0.14 | 0.64 |
| pXyl-LNH-4# | Pzpk-pXyl-20190821-hyg-4# | Diploid, p*XYL-CltLS1-NPPS-HMGR-Hyg-*Thsp, Hyg^R^ | Glucose | 0.19 | 0.08 | 1.90 |
| pXyl-CltLS1-np11 | Pzpk-pXyl-CltLS1-Ble-np11 | *MAT A1,* p*XYL-CltLS1-Ble-*Thsp, Ble^R^ | Glucose | 28.85 | 25.91 | 56.00 |
| pXyl-CltLS2-np11 | Pzpk-pXyl-CltLS2-Ble-np11 | *MAT A1,* p*XYL-CltLS2-Ble-*Thsp, Ble^R^ | Glucose | 0.0 | 0.00 | 0.00 |
| pXyl-CstLS-np11 | Pzpk-pXyl-CstLS-Ble-np11 | *MAT A1,* p*XYL-CstLS-Ble-*Thsp, Ble^R^ | Glucose | 0.54 | 0.00 | 6.67 |
| pXyl-ChtLS-np11 | Pzpk-pXyl-ChtLS-Ble-np11 | *MAT A1,* p*XYL-ChtLS-Ble-*Thsp, Ble^R^ | Glucose | 0.00 | 0.00 | 0.00 |
| pXyl-PftLS-np11 | Pzpk-pXyl-PftLS-Ble-np11 | *MAT A1,* p*XYL-PftLS-Ble-*Thsp, Ble^R^ | Glucose | 0.00 | 0.00 | 0.00 |
| pXyl-ArtLS-np11 | Pzpk-pXyl-ArtLS-Ble-np11 | *MAT A1,* p*XYL-ArtLS-Ble-*Thsp, Ble^R^ | Glucose | 2.21 | 0.00 | 11.55 |
| pXyl-VvtLS-np11 | Pzpk-pXyl-VvtLS-Ble-np11 | *MAT A1,* p*XYL-VvtLS-Ble-*Thsp, Ble^R^ | Glucose | 0.00 | 0.00 | 0.00 |
| pXyl-MetLS-np11 | Pzpk-pXyl-MetLS-Ble-np11 | *MAT A1,* p*XYL-MetLS-Ble-*Thsp, Ble^R^ | Glucose | 0.00 | 0.00 | 0.00 |
| pXyl-LNH-ΔCrt-np11 | Pzpk-PXyl-20190821-Ntc-ΔCrt-np11 | *MAT A1 ΔCrt,*  p*XYL-CltLS1-NPPS-HMGR-*ΔCrt*-Ntc-Hyg-*Thsp, Hyg^R^ Ntc^R^ | Glucose | 6.69 | 4.17 | 37.71 |
| pXyl-LS1-LNH-ΔCrt-np11 | Pzpk-PXyl-CltLS1-20190821-Ntc-ΔCrt-np11-16# | *MAT A1 ΔCrt,*  p*XYL-2*CltLS1-NPPS-HMGR-*ΔCrt*-Ble-Ntc-Hyg-*Thsp, Hyg^R^ Ntc^R^ Ble^R^ | Glucose | 14.22 | 14.53 | 28.58 |
| pXyl-LN-LNH-ΔCrt-np11 | Pzpk-PXyl-LN-201908212-Ntc-ΔCrt-np11-16# | *MAT A1 ΔCrt,*  p*XYL-2*CltLS1-2*NPPS-HMGR-*ΔCrt*-Ble-Ntc-Hyg-*Thsp, Hyg^R^ Ntc^R^ Ble^R^ | Glucose | 15.45 | 12.10 | 39.05 |
| pXyl-KSE-LNH-ΔCrt-np11 | Pzpk-PXyl-KSE-201908212-Ntc-ΔCrt-np11-16# | *MAT A1 ΔCrt,*  p*XYL-CltLS1-NPPS-HMGR-EfMvaE-EfMvaS-MmMK-*ΔCrt*-Ble-Ntc-Hyg-*Thsp, Hyg^R^ Ntc^R^ Ble^R^ | Glucose | 54.43 | 52.33 | 107.23 |
| pXyl-L--NH-ΔCrt-np11 | Pzpk-PXyl-LS1-GGGS-NPPS-HMGR-Ntc-ΔCrt-np11 | *MAT A1 ΔCrt,*  p*XYL-CltLS1-GGGS-NPPS-HMGR-*ΔCrt*-Ble-Ntc-Hyg-*Thsp, Hyg^R^ Ntc^R^ Ble^R^ | Glucose | 5.83 | 3.75 | 25.36 |
| pXyl-N--LH-ΔCrt-np11 | Pzpk-PXyl-NPPS-GGGS-LS1-HMGR-Ntc-ΔCrt-np11 | *MAT A1 ΔCrt,*  p*XYL-NPPS-GGGS-CltLS1-HMGR-*ΔCrt*-Ble-Ntc-Hyg-*Thsp, Hyg^R^ Ntc^R^ Ble^R^ | Glucose | 23.43 | 12.35 | 139.74 |
| pXyl-KSE-N--LH-ΔCrt-np11 | Pzpk-PXyl-KSE-NPPS-GGGS-LS1-HMGR-Ntc-ΔCrt-np11 | *MAT A1 ΔCrt,*  p*XYL-NPPS-GGGS-CltLS1-HMGR-EfMvaE-EfMvaS-MmMK-*ΔCrt*-Ble-Ntc-Hyg-*Thsp, Hyg^R^ Ntc^R^ Ble^R^ | Glucose | 23.43 | 12.35 | 393.48 |
| pXyl-PrIDI-N--LH-ΔCrt-np11 | Pzpk-PXyl-PrIDI-NPPS-GGGS-LS1-HMGR-Ntc-ΔCrt-np11 | *MAT A1 ΔCrt,*  p*XYL-PrIDI-NPPS-GGGS-CltLS1-HMGR-*ΔCrt*-Ble-Ntc-Hyg-*Thsp, Hyg^R^ Ntc^R^ Ble^R^ | Glucose | 18.64 | 17.20 | 66.95 |
| pXyl-MmMK-KSE-N--LH-ΔCrt-np11 | Pzpk-PXyl-KSE-NPPS-GGGS-LS1-HMGR-Ntc-ΔCrt-np11 | *MAT A1 ΔCrt,*  p*XYL-MmMK-NPPS-GGGS-CltLS1-HMGR-*ΔCrt*-Ble-Ntc-Hyg-*Thsp, Hyg^R^ Ntc^R^ Ble^R^ | Glucose | 35.74 | 23.82 | 110.45 |
| pXyl-(-)SGH-ΔCrt-np11 | Pzpk-PXyl-(-)LS-GPPS-HMGR-Ntc-ΔCrt-np11 | *MAT A1 ΔCrt,*  p*XYL-MmMK-(-)LS-GPPS-HMGR-*ΔCrt*-Ble-Ntc-Hyg-*Thsp, Hyg^R^ Ntc^R^ | Glucose | 7.48 | 14.78 | 16.73 |

Table S2. Protein sequence and source in this study.

| Protein name | Sequence | Source |
| --- | --- | --- |
| *CltLS1* | DRRSANYQPSIWDHDFLQSLNSNYTDEAYKRRAEELRGKVKIAIKDVIEPLDQLELIDNLQRLGLAHRFETEIRNILNNIYNNNKDYNWRKENLYATSLEFRLLRQHGYPVSQEVFNGFKDDQGGFICDDFKGILSLHEASYYSLEGESIMEEAWQFTSKHLKEVMISKNMEEDVFVAEQAKRALELPLHWKVPMLEARWFIHIYERREDKNHLLLELAKMEFNTLQAIYQEELKEISGWWKDTGLGEKLSFARNRLVASFLWSMGIAFEPQFAYCRRVLTISIALITVIDDIYDVYGTLDELEIFTDAVERWDINYALKHLPGYMKMCFLALYNFVNEFAYYVLKQQDFDLLLSIKNAWLGLIQAYLVEAKWYHSKYTPKLEEYLENGLVSITGPLIITISYLSGTNPIIKKELEFLESNPDIVHWSSKIFRLQDDLGTSSDEIQRGDVPKSIQCYMHETGASEEVARQHIKDMMRQMWKKVNAYTADKDSPLTGTTTEFLLNLVRMSHFMYLHGDGHGVQNQETIDVGFTLLFQPIPLEDKHMAFTASPGTKG | *Citrus limon* |
| *CltLS2* | DRRSANYQPSIWDHDFLQSLNSNYTDETYRRRAEELKGKVKIAIKDVTEPLDQLELIDNLQRLGLAYRFETEIRNILHNIYNNNKDYVWRKENLYATSLEFRLLRQHGYPVSQEVFNGFKDDQGGFIFDDFKGILSLHEASYYSLEGESIMEEAWQFTSKHLKEVMISKSMEEDVFVAEQAKRALELPLHWKVPMLEARWFIHVYEKREDKNHLLLELAKMEFNTLQAIYQEELKEISGWWKDTGLGEKLSFARNRLVASFLWSMGIAFEPQFAYCRRVLTISIALITVIDDIYDVYGTLDELEIFTDAVARWDINYALKHLPGYMKMCFLALYNFVNEFAYYVLKQQDFDMLLSIKNAWLGLIQAYLVEAKWYHSKYTPKLEEYLENGLVSITGPLIIAISYLSGTNPIIKKELEFLESNPDIVHWSSKIFRLQDDLGTSSDEIQRGDVPKSIQCYMHETGASEEVAREHIKDMMRQMWKKVNAYTADKDSPLTRTTTEFLLNLVRMSHFMYLHGDGHGVQNQETIDVGFTLLFQPIPLEDKDMAFTASPGTKG | *Citrus limon* |
| *CstLS* | DRRSANYQPSIWDHDFLQSLNSNYTDETYKRRAEELKGKVKTAIKDVTEPLDQLELIDNLQRLGLAYHFEPEIRNILRNIHNHNKDYNWRKENLYATSLEFRLLRQHGYPVSQEVFSGFKDDKVGFICDDFKGILSLHEASYYSLEGESIMEEAWQFTSKHLKEMMITSNSKEEDVFVAEQAKRALELPLHWKAPMLEARWFIHVYEKREDKNHLLLELAKLEFNTLQAIYQEELKDISGWWKDTGLGEKLSFARNRLVASFLWSMGIAFEPQFAYCRRVLTISIALITVIDDIYDVYGTLDELEIFTDAVARWDINYALKHLPGYMKMCFLALYNFVNEFAYYVLKQQDFDMLLSIKHAWLGLIQAYLVEAKWYHSKYTPKLEEYLENGLVSITGPLIITISYLSGTNPIIKKELEFLESNPDIVHWSSKIFRLQDDLGTSSDEIQRGDVPKSIQCYMHETGASEEVAREHIKDMMRQMWKKVNAYTADKDSPLTRTTAEFLLNLVRMSHFMYLHGDGHGVQNQETIDVGFTLLFQPIPLEDKDMAFTASPGTKG | *Citrus sinensis* |
| *ChtLS* | TRRTGNHHGNLWDDDFIQSLPKLPYDAPEYRERADRLVGEVKNMFNAVRAADSSSQNILRLLEMVDKVERLGIGRHFETEIAEALDYVYRFWNDISSKDLNTAALGLRILRLHRYPVSSDVLEQFKEKDGHFLCCTTQLEEEIKSILNLFRASLIAFPNEKIMDEAKAFSTMYLKQVFQKSHILGTHLLKEITFNLEYGWRTNLPRLEARNYMDIYGENSSWLMDMDNKNILYLAKLDFNILQSLYRPELQMISRWWKDSSLYKLDFSRHRHIEYLFQGCAITGEPKHSGFRIDIAKYSTLATIIDDIYDTYGSIEELKHFTEVFKRWDSSPPDYLPEYMKIAYSALYDGINKSAQEAVQIQGRDTLHNARNAWDDYLDAVMQEAKWNSIGHMPNLKEFLENGRVSSGTRVITLQALLRLEALQESELQKIDHPSKFNYLFGLTLRLRGDTRTFKAEANRGEVTSSIACYLKEHPESTEKDALKYLQFMLDENLKELNLEYLKNDGVPICIKDFAYDMSRCFEVFYKERDGFSISTKDMKNHVERILIEPVEM | *Chamaecyparis obtuse* |
| *PftLS* | QRRSGNYSPSFWNADYILSLNSHYKDKSHMKRAGELIVQVKMVMGKETDPVVQLELIDDLQKLALSHHVEKEIKEILFKISTYDHKIMVERDLYSTALAFRLLRQYGFKVPQEVFDCFKNDNGEFKRSLSSDTKGLLQLYEASFLLTEGEMTLELAREFATKSLQEKLNEKTIDDDDDADTNLISCVRHSLDIPIHWRIQRPNASWWIDAYKRRSHMNPLVLELAKLDLNIFQAQFQQELKQDLGWWKNTCLAEKLPFVRDRLVECYFWCTGIIQPLVECYFWCTGIIQPLQHENARVTLAKVNALITTLDDIYDVYGTLEELELFTEAIRRWDVSSIDHLPNYMQLCFLALNNFVDDTAYDVMKEKDINIIPYLRKSWLDLAETYLVEAKWFYSGHKPNLEEYLNNAWISISGPVMLCHVFFRVTDSITRETVESLFKYHDLIRYSSTILRLADDLGTSLEEVSRGDVPKSIQCYMNDNNASEEEARRHIRWLIAETWKKINEEVWSVDSPFCKDFIACAADMGRMAQFMYHNGDGHGIQNPQIHQQMTDILF | *perilla frutescens* |
| *ArtLS* | ERRSGNYSPSRWDVDFIQSLNSDYQEERHTRRASELITQVKMLMEKETTDPIRQLELIDDLQRLGLSDHFQNEFKEILNTIYLDNKYYNINIMREESRDLYSTALAFRLLREHGFQVAQEVFECFKNEEGDFKASLIDDTRGLLQLYEASFLFKEGENTLEIAREFTTKILQEKLKGDEIDDNLLSSIRYSLEIPNYWSVVRPNVSVWIDEYRKRSDMNPVVLELAILDANIVQAQLQLELKESLRWWRNTCFVEKLPFARDRLIESYFWSTGMVEPRQHANARIIMAKVIALITVMDDIYDVYGTLEELEQFTEAFRRWDVSSIDQLPTYMQLCFLAINNFVDDTAYNVLKESGVNVMTYLRKSWVDQAENYLMESKWYYSGHKPSLEEYLENSWISVSGPCVLTHEFFGVTDSLAKDTLDSLYEYHDIVRWSSYLLRLADDLGTSVEEVSRGDVPKSIQCYMHDNDASEEEARQHIKGLIREMWKKMNVERVSEDSPFCRDFIRCCEDLGRMAQFMYHYGDGHGTQHPKIHQQIAACLFQPFA | *Agastache rugose* |
| *VvtLS* | VRRTASYQPSIWDHDYIRSLTSDYVGETYTRQLEKLKGDVKIMLGQVGEPLHQLELIDTLQRLGIHYHFGEEIKRILHSIYNNYNRNDTWKNGDLYATALEFRLLRQHGYHVPQDVFHIFINKMGTVKPWLNEDIKGILCLYEASYLSVEGENILEEARDFTRNFLEEYLERTVDQNDLTAIINHAMELPLHWRMLRLEARWFIDENVDKLISLITTIDDVYDVYGTLDELELFTDAVDRWDTNAMEQLPQYMKICFLALYNFTNETAYDVLKEHDLNIISYLRNAWADITKSQLVEAKWYHEGYKPSLQEYINNAWISVSGPLTLVHAYFFITNPMTEEALGCLERFRDIIRWSSTIFRLSDDLGTSSDELKRGDVPKSIQCYMYETSASEDDARKYIGFLIDETWKKMNEERNLNSPFSQTFIGMAMDIPRMAQCIYLYRDGYGVQDRETKDHVKTLFIEPISLI | *Vitis vinifera* |
| *MetLS* | ERRSGNYNPSRWDVDFIQTLHSDYKDEKHARRASELVTLVKMELEKETDQIRQLELIDDLQRMGLSDHFQNEFKEILSSVYLDHGYYKNPDPKEERDLYSTSLAFRLLREHGFQVAQEVFDSFKNEEGEFKESLSDDTRGLLQLYEASFLLTEGETTLESAREFATKFLEERVNEGGGDENLLTRIAYSLEIPLHWRIKRPNAPVWIDSYRKRPNMNPVVLDLAILDLNIVQAHFQQELKESFRWWRNTGFVEKLPFARDRLVECYFWNTGIIEPRQHASARIMMGKVNALITVIDDIYDVYGTLEELEHFTDLIRRWDIDSIDQLPDYMQLCFLALNNFVDETSYDVMKEKGVNVIPYLRQSWVDLADKYMVEARWFYGGHKPSLEEYLENSWMSISGPCMLTHIFFRVTDSFTKETVDSLYKYHDLVRWSSFVLRLADDLGTSVEEVSRGDVPKSLQCYMSDYNASEAEARKHVKWLIAEVWKKMNAERVSKDSPFGKDFIGCAVDLGRMAQLMYHNGDGHGTQHPIIHQQMTATLFEPFA | *Mentha×piperita* |
| *IDI* | MSMPNIVPPAEVRTEGLSLEEYDEEQVRLMEERCILVNPDDVAYGEASKKTCHLMSNINAPKDLLHRAFSVFLFRPSDGALLLQRRADEKITFPGMWTNTCCSHPLSIKGEVKEENQIGVRRAASRKLEHELGVPTSSTPPDSFTYLTRIHYLAPSDGLWGEHEIDYILFSTTPTEHTGNPNEVSDTRYVTKPELQAMFEDESNSFTPWFKLIARDFLFGWWDQLLARRNEKGEVDAKSLEDLSDNKVWKM | *Phaffia rhodozyma* |
| *NPPS* | RGLNKISCSLNLQTEKLCYEDNDNDLDEELMPKHIALIMDGNRRWAKDKGLEVYEGHKHIIPKLKEICDISSKLGIQIITAFAFSTENWKRSKEEVDFLLQMFEEIYDEFSRSGVRVSIIGCKSDLPMTLQKCIALTEETTKGNKGLHLVIALNYGGYYDILQATKSIVNKAMNGLLDVEDINKNLFDQELESKCPNPDLLIRTGGEQRVSNFLLWQLAYTEFYFTNTLFPDFGEEDLKEAIMNFQQRHRRFGGHTY | *Solanum lycopersicum* |
| *HMGR* | GSGEGRGSLLTCGDVEENPGPILIRTRKALNGAPSSSTLTVPSTDEVTAPQLKLSPSTVALVSQNGIPDTPRDLDTCVKIFNGGEGAMLLNDEEIITLVQKGKLAAYALEKLLKDYVRAVSIRRALISRASARKTLEASDLPFLHFDYSRVMGQCCENVVGYMPIPVGIAGPLRIDGNVLPIPMATTEGALVASTSRGCKALNVSGGVTTVVTQDAMTRGPALDFPSVIMCAAAKRWVDSDEGSNILKAAFNSTSRFARLKSLKTAMAGRTLFVRFATQTGDAMGMNMISKGCERALDVMMTEHFPEMKIASLSGNYCTDKKPAAINWIEGRGKSVVAEGIIPGEAVKSILKTTVSDLVRLNITKNLIGSAMAGSVGGNNAHASNILTAIYLATGQDPAQNVESSNCMTLMEAINDGKDLLITCSMPSIEVGTVGGGTILLPQAAMLDMLGVKGPHPTSPGQNAQQLARVVCAAVMAGELSLMSALAAGSLVQSHLAHNRSAPATPAAQTPQIGSRAATPVLNGTQRLAPLTVTKGKD | *R. toruloides* |
| *MVK* | MVSCSAPGKIYLFGEHAVVYGETAIACAVELRTRVRAELNDSITIQSQIGRTGLDFEKHPYVSAVIEKMRKSIPINGVFLTVDSDIPVGSGLGSSAAVTIASIGALNELFGFGLSLQEIAKLGHEIEIKVQGAASPTDTYVSTFGGVVTIPERRKLKTPDCGIVIGDTGVFSSTKELVANVRQLRESYPDLIEPLMTSIGKISRIGEQLVLSGDYASIGRLMNVNQGLLDALGVNILELSQLIYSARAAGAFGAKITGAGGGGCMVALTAPEKCNQVAEAVAGAGGKVTITKPTEQGLKVDGS | *Methanosarcina mazei* |
| *MvaS* | ATNFSLLKQAGDVEENPGPMTIGIDKISFFVPPYYIDMTALAEARNVDPGKFHIGIGQDQMAVNPISQDIVTFAANAAEAILTKEDKEAIDMVIVGTESSIDESKAAAVVLHRLMGIQPFARSFEIKEACYGATAGLQLAKNHVALHPDKKVLVVAADIAKYGLNSGGEPTQGAGAVAMLVASEPRILALKEDNVMLTQDIYDFWRPTGHPYPMVDGPLSNETYIQSFAQVWDEHKKRTGLDFADYDALAFHIPYTKMGKKALLAKISDQTEAEQERILARYEESIIYSRRVGNLYTGSLYLGLISLLENATTLTAGNQIGLFSYGSGAVAEFFTGELVAGYQNHLQKETHLALLDNRTELSIAEYEAMFAETLDTDIDQTLEDELKYSISAINNTVRSYRN | *Enterococcus faecalis* |
| *MvaE* | EGRGSLLTCGDVEENPGPMKTVVIIDALRTPIGKYKGSLSQVSAVDLGTHVTTQLLKRHSTISEEIDQVIFGNVLQAGNGQNPARQIAINSGLSHEIPAMTVNEVCGSGMKAVILAKQLIQLGEAEVLIAGGIENMSQAPKLQRFNYETESYDAPFSSMMYDGLTDAFSGQAMGLTAENVAEKYHVTREEQDQFSVHSQLKAAQAQAEGIFADEIAPLEVSGTLVEKDEGIRPNSSVEKLGTLKTVFKEDGTVTAGNASTINDGASALIIASQEYAEAHGLPYLAIIRDSVEVGIDPAYMGISPIKAIQKLLARNQLTTEEIDLYEINEAFAATSIVVQRELALPEEKVNIYGGGISLGHAIGATGARLLTSLSYQLNQKEKKYGVASLCIGGGLGLAMLLERPQQKKNSRFYQMSPEERLASLLNEGQISADTKKEFENTALSSQIANHMIENQISETEVPMGVGLHLTVDETDYLVPMATEEPSVIAALSNGAKIAQGFKTVNQQRLMRGQIVFYDVADPESLIDKLQVREAEVFQQAELSYPSIVKRGGGLRDLQYRTFDESFVSVDFLVDVKDAMGANIVNAMLEGVAELFREWFAEQKILFSILSNYATESVVTMKTAIPVSRLSKGSNGREIAEKIVLASRYASLDPYRAVTHNKGIMNGIEAVVLATGNDTRAVSASCHAFAVKEGRYQGLTSWTLDGEQLIGEISVPLALATVGGATKVLPKSQAAADLLAVTDAKELSRVVAAVGLAQNLAALRALVSEGIQKGHMALQARSLAMTVGATGKEVEAVAQQLKRQKTMNQDRAMAILNDLRKQ | *Enterococcus faecalis* |
|  |  |  |

Table S3. Primer sequence used in vector preparation.

| Primer name | Sequence (5’ to 3’) |
| --- | --- |
| LS1-F | CCGGATATCATGGACCGCCGCTCGG |
| Overlap-GGGS-LS1-R | AGATCTTGTTGAGGCCGCGCGCCGAGCCGCCGCCCTTGGTGCCTGGCGAGG |
| Overlap-NPPS-HMG-F | CACCGCCTCGCCAGGCACCAAGGGCGGCGGCTCGGCGCGCGGCCTCAACAAGA |
| tHMGR-SpeI-R | GGACTAGTCTAGTGGTGGTGGTGGTGG |
| IDIRT-F | GGTGTGAGTGGTGCAAGACAT |
| IDIRT-R | TCACATGCGGATGATGCTGC |
| tHMGR-ECoRV-F | GATATCATGATCCTCATCCG |
| XYL-XbaⅠ-F | GGCTCTAGATGTCCGTATTCTACATC |
| XYL-EcoRⅤ-R | GCTTGATATCGGCGTGTATTCTGCGTG |
| LAD-XbaⅠ-F | GGTCTAGAGAGTCAAGGTGAGGTTG |
| LAD-EcoRⅤ-R | CAAGATATCCCTGGCGGGCGTGGAG |
| CltLS1-EcoRⅤ-F | TCTAGAGATATCATGGACCGCCGCTC |
| CltLS2-SpeⅠ-R | GGACTAGTCTAATGGTGATGGTGG |
| PfLS-EcoRⅤ-F | TCTAGAGATATCATGCAGCGCCGCTC |
| NPPS-LS-EcoRⅤ-F | TCTAGAGATATCATGGAACGCCGCTC |
| MpLs-EcoRⅤ-F | TCTAGAGATATCATGGAGCGCCGCTC |
| NPPS-EcoRⅤ Fusion-F | CTTAGATATCATGTCGGCGCGCGG |
| NPPS Fusion-R | GTAGTTGCCCGAGCGGCGTTCAGGGCCCGGGTTCTCCTCGACG |
| LS Fusion-F | CGAGGAGAACCCGGGCCCTGAACGCCGCTCGGGCAACTACTC |
| LS-Spe Ⅰ Fusion-R | GGACTAGTCTAGTGGTGGTGGTGG |

Table S4. Plasmids used in this study.

| **Plasmids** | **Source** |
| --- | --- |
| Pzpk-pPGK-Hyg-Tnos-pGPD-*CltLS1*-Thsp | This study |
| Pzpk-pPGK-Hyg-Tnos-pGPD-*CltLS1*-*NPPS*-Thsp | This study |
| Pzpk-pPGK-Hyg-Tnos-pGPD*-CltLS1-NPPS-HMGR-*Thsp | This study |
| Pzpk-pPGK-Hyg-Tnos-pAra*-CltLS1-NPPS-HMGR-*Thsp | This study |
| Pzpk-pPGK-Hyg-Tnos-pXyl*-CltLS1-NPPS-HMGR-*Thsp | This study |
| Pzpk-pPGK-Ble-Tnos-pXyl*-CltLS1-*Thsp | This study |
| Pzpk-pPGK-Ble-Tnos-pXyl*-CltLS1--NPPS-*Thsp | This study |
| Pzpk-pPGK-Ble-Tnos-pXyl*-CltLS1--NPPS-HMGR-*Thsp | This study |
| Pzpk-pPGK-Ble-Tnos-pXyl*-CltLS2--NPPS-HMGR-*Thsp | This study |
| Pzpk-pPGK-Ble-Tnos-pXyl*-CstLS--NPPS-HMGR-*Thsp | This study |
| Pzpk-pPGK-Ble-Tnos-pXyl*-ChtLS--NPPS-HMGR-*Thsp | This study |
| Pzpk-pPGK-Ble-Tnos-pXyl*-PftLS--NPPS-HMGR-*Thsp | This study |
| Pzpk-pPGK-Ble-Tnos-pXyl*-ArtLS--NPPS-HMGR-*Thsp | This study |
| Pzpk-pPGK-Ble-Tnos-pXyl*-VvtLS--NPPS-HMGR-*Thsp | This study |
| Pzpk-pPGK-Ble-Tnos-pXyl*-MetLS--NPPS-HMGR-*Thsp | This study |
| Pzpk-pPGK-Ntc-Tnos-pXyl*-CltLS1-NPPS-HMGR-*Thsp | This study |
| Pzpk-pPGK-Ntc-Tnos-pXyl*-EfMvaE-EfMvaS-MmMK-*Thsp | This study |
| Pzpk-pPGK-Ble-Tnos-pXyl-*CltLS1-GGGS-NPPS-HMGR-*Thsp | This study |
| Pzpk-pPGK-Ble-Tnos-pXyl-*NPPS-GGGS-CltLS1-HMGR-*Thsp | This study |
| Pzpk-pPGK-*IDIRt*-Ble-Tnos-pXyl-*NPPS-GGGS-CltLS1-HMGR-*Thsp | This study |
| Pzpk-pPGK-Ble-Tnos-pXyl-Pr*IDI-NPPS-GGGS-CltLS1-HMGR-*Thsp | This study |
| Pzpk-pPGK-Ntc-Tnos-pXyl*-MmMK-EfMvaE-EfMvaS-MmMK-*Thsp | This study |
| Pzpk-pPGK-Ble-Tnos-pXyl-(-)LS-GPPS*-HMGR-*Thsp | This study |
